# Transcription and phosphorylation of CATALASE2 contribute to cold stress tolerance in *Hevea brasiliensis*

**DOI:** 10.1111/tpj.71097

**Published:** 2026-08-28

**Authors:** Ru‐Feng Song, Wei Wen, Lin Li, Huan Yang, Chaorong Tang, Wen‐Cheng Liu, Hong‐Mei Yuan

**Affiliations:** ^1^ National Key Laboratory of Tropical Crop Breeding Sanya Institute of Breeding and Multiplication, School of Tropical Agriculture and Forestry, Hainan University Sanya 572025 China; ^2^ State Key Laboratory of Crop Stress Adaptation and Improvement School of Life Sciences, Henan University Kaifeng 475004 China; ^3^ Sanya Institute, Henan University Sanya 572025 China

**Keywords:** *Hevea brasiliensis*, cold stress, catalase, H_2_O_2_, transcription, SnRK2.6, phosphorylation

## Abstract

Despite the economic importance of the rubber tree (*Hevea brasiliensis*) and its sensitivity to cold stress, the molecular mechanisms that govern its cold response are still largely unknown. Here, we identified *H. brasiliensis* CATALASE2 (HbCAT2) as a factor associated with cold tolerance in this species, likely through reducing excess H_2_O_2_. Cold stress significantly induced *HbCAT2* transcription and catalase activity in *H. brasiliensis*, and overexpression of *HbCAT2* in both *H. brasiliensis* and Arabidopsis enhanced cold tolerance and reduced H_2_O_2_ accumulation. Furthermore, we found that HbCBF1, a key transcription factor in plant cold signaling, could directly bind to and activate *HbCAT2*, and overexpression of *HbCAT2* was able to alleviate the cold hypersensitivity of the Arabidopsis *cbf123* triple mutant. Additionally, the protein kinase HbSnRK2.6A interacted with and phosphorylated HbCAT2 to stimulate its catalase activity. Genetic evidence showed that HbSnRK2.6A‐mediated cold tolerance is associated with CAT2 function. Together, our findings are consistent with a dual regulatory module in which HbCBF1‐mediated *HbCAT2* transcription and HbSnRK2.6A‐mediated phosphorylation of HbCAT2 may contribute to enhancing cold tolerance by preventing H_2_O_2_ overaccumulation in *H. brasiliensis*.

## Significance Statement

Despite the rubber tree's economic importance and sensitivity to cold stress, the molecular mechanisms governing its cold tolerance remain largely unknown. This study reveals a dual regulatory module in which HbCBF1 mediated HbCAT2 transcription and HbSnRK2.6A‐mediated HbCAT2 phosphorylation synergistically enhance cold tolerance by preventing H_2_O_2_ overaccumulation in *Hevea brasiliensis*.

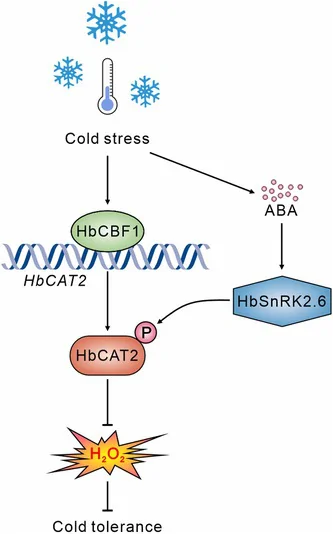

## INTRODUCTION

The rubber tree (*Hevea brasiliensis*) is an economically important tropical species. Its latex is the sole commercial source of natural rubber, an essential industrial raw material (Van Beilen & Poirier, 2007). As a typical tropical rainforest tree, warm temperatures sustain the growth and development of *H. brasiliensis*, while low temperatures restrict its growth, limiting its geographical distribution and reducing latex yield (Chen et al., 2019; Cheng et al., 2018; Mao et al., 2023).

To survive cold temperatures, plants have evolved sophisticated mechanisms to protect themselves (Ding & Yang, 2022; Guo et al., 2018; Raza et al., 2023). Cold acclimatization, which involves exposing plants to non‐freezing temperatures, enhances their cold tolerance (Thomashow, 1999). During this process, C‐repeat (CRT)‐binding factors (CBFs) activate cold‐regulated (*COR*) genes to increase cold resistance (Chinnusamy et al., 2003). Two MYC‐type basic helix–loop–helix (bHLH) transcription factors, Inducer of CBF Expression (ICE) 1 and ICE2, bind to the promoter of *CBF* genes, inducing their expression and thus enhancing cold tolerance (Chinnusamy et al., 2003; Fursova et al., 2009). Cold‐activated protein kinases, such as Open Stomata 1 (OST1), MAP Kinase (MPK) 3/6, and Brassinosteroid Insensitive 2 (BIN2), are involved in cold responses through the ICE‐CBF‐COR pathway (Ding et al., 2015; Ye et al., 2019; Zhao et al., 2017). Besides ICE1, other transcription factors like CAMTA3, LONG HYPOCOTYL 5 (HY5), MYB15, and Brassinazole‐Resistant 1 (BZR1) also promote cold tolerance by activating CBF gene expression (Agarwal et al., 2006; Doherty et al., 2009; Li et al., 2017, 2021).

Although the components and mechanisms involved in cold stress response and tolerance have been extensively investigated in the model plant *Arabidopsis thaliana*, the cold signaling pathway and factors in crop plants such as *H. brasiliensis* remain poorly understood. We recently reported that HbICE1 and HbSnRK2.6 play a positive role whereas HbEIN3, a transcription factor in ethylene signaling, plays a negative role in cold stress tolerance in *H. brasiliensis* (Wang et al., 2021; Yuan, Sheng, et al., 2017; Zeng et al., 2025). Similarly, ZmICE1 in maize is crucial for cold tolerance by regulating amino acid metabolism and reactive oxygen species homeostasis (Jiang et al., 2022). Besides, the transcription factor HbCBF1 was identified as a functional transcriptional activation factor that can promote cold tolerance in *H. brasiliensis* (Cheng et al., 2015).

Abscisic acid (ABA) is a crucial stress phytohormone acting in plant responses to various environmental stresses, including drought, osmotic, high salinity, high temperature, and heavy metals (Lu et al., 2026; Vishwakarma et al., 2017; Yoshida et al., 2014). Under adverse conditions, ABA level increases and ABA is perceived by the receptors PYRABACTIN RESISTANCE PROTEINS/PYR‐LIKE PROTEINS/REGULATORY COMPONENTS OF ABA RECEPTOR (PYR/PYL/RCAR), relieving PHOSPHATASE2C (PP2C)‐mediated inhibition of SNF1‐RELATED PROTEIN KINASE2 (SnRK2) kinases (Ma et al., 2009; Park et al., 2009). Activated SnRK2 kinases phosphorylate downstream ABA‐responsive transcription factors to modulate the expression of stress‐ or ABA‐related genes, or phosphorylate ion channels to control the exchanges of ions (Fujii et al., 2009; Guo et al., 2022; Yoshida et al., 2014). In addition to these ABA‐related transcription factors or ion channels, SnRK2 kinases also interact with and phosphorylate other functional proteins, such as ICE1, a critical factor in plant cold signaling pathway. It was reported that SnRK2.6 could stabilize ICE1 protein to enhance the expression of CBFs through the phosphorylation in Arabidopsis (Ding et al., 2015). Intriguingly, cold‐activated SnRK2.6 kinase activity and the phosphorylation of ICE1 were reported to be independent of ABA levels (Ding et al., 2015). However, ABA and SnRK2.6 involved in *H. brasiliensis* resistance to cold stress remains largely unknown.

ROS are oxygen‐containing molecules with higher chemical activity than regular oxygen, including superoxide O_2_^•−^, hydroxyl OH^•^, hydrogen peroxide (H_2_O_2_) and singlet oxygen ^1^O_2_ (Liu et al., 2026; Song et al., 2026). ROS are naturally formed as byproducts during essential life processes while many environmental stresses including low temperatures can induce ROS overaccumulation, leading to oxidative stress (Baxter et al., 2014; Czarnocka & Karpiński, 2018; Wang et al., 2024). We previously reported that cold stress can significantly trigger the production and accumulation of H_2_O_2_ in *A. thaliana* and *H. brasiliensis* (Liu et al., 2022; Yuan, Sheng, et al., 2017), and overexpression of *HbICE1* could reduce the accumulation of ROS compared to the wild‐type under cold stress conditions  (Yuan, Sheng, et al., 2017), suggesting a tight correlation of antioxidant activity with the cold stress tolerance in *H. brasiliensis*.

Long‐term evolution has endowed plants with complex and precise antioxidant systems, including antioxidant enzymes such as catalase (CAT), ascorbate peroxidase (APX), superoxide dismutase (SOD), and non‐enzymatic antioxidants such as glutathione, ascorbate, anthocyanin, and melatonin (Janků et al., 2019; Smirnoff & Arnaud, 2019; Wang et al., 2024; Yang et al., 2021). CAT plays a vital role in plant stress resistance by scavenging excess H_2_O_2_. Previous studies revealed that the mutant plants with reduced CAT activity were sensitive to salt stress, drought stress, heat stress, heavy metals, and necrotrophic pathogen infection (Li et al., 2015; Liu et al., 2017; Ono et al., 2021; Tian et al., 2023; Yang et al., 2023; Yuan, Liu, & Lu, 2017; Zhang et al., 2020), while its role in low temperature stress remains largely unknown.

In this study, we identified HbCAT2 as a potential positive regulator of cold tolerance in *H. brasiliensis*. We found that cold stress induces *HbCAT2* transcription to increase catalase activity, while overexpression of *HbCAT2* reduces H_2_O_2_ accumulation and enhances cold tolerance in both wild‐type *H. brasiliensis* and Arabidopsis. On the one hand, the transcription factor HbCBF1 directly binds to and activates *HbCAT2*, which may contribute to enhanced plant cold tolerance; on the other hand, HbSnRK2.6A kinase physically interacts with and phosphorylates HbCAT2 to stimulate its catalase activity, potentially further promoting plant cold tolerance. Our study demonstrates that both the transcriptional and post‐translational regulation of HbCAT2 may enhance cold tolerance through promoting catalase activity and reducing excess H_2_O_2_ accumulation in *H. brasiliensis*.

## RESULTS

### Catalase is required for the cold tolerance in *H. brasiliensis*

**Figure 1:**
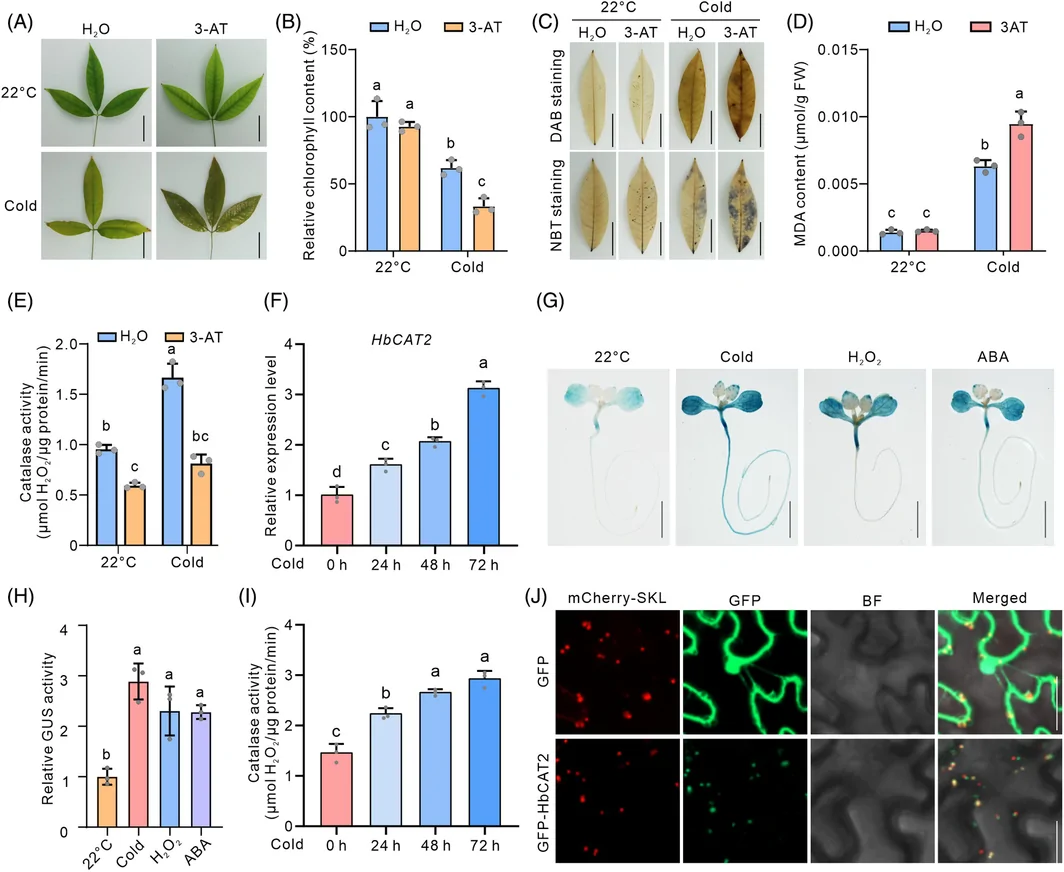
Catalase activity is required for *Hevea brasiliensis* cold tolerance. (A, B) Leaf phenotype (A) of the *H. brasiliensis* seedlings treated with cold in the presence of H_2_O or 3‐AT. The *H. brasiliensis* seedlings were treated with H_2_O or 5 mM 3‐AT under 22°C or 10°C for 7 days, and then the chlorophyll content (B) was measured. Data are means ± SD (*n* = 3). Bars with different letters indicate significant differences at *P* < 0.05, determined using anova with Tukey's multiple comparison test. Bars = 5 cm. (C) 3,3‐Diaminobenzidine (DAB) and nitroblue tetrazolium (NBT) staining images of the *H. brasiliensis* seedling leaves shown in (A). Bars = 5 cm. (D) Malondialdehyde (MDA) content in *H. brasiliensis* seedling leaves treated with 22°C or 10°C for 3 days. Data are means ± SD (*n* = 3). Bars with different letters indicate significant differences at *P* < 0.05, determined using anova with Tukey's multiple comparison test. (E) Relative catalase activities of the *H. brasiliensis* seedling leaves shown in (A). (F) Expression levels of *HbCAT2* in *H. brasiliensis* response to cold temperatures. The rubber tree seedlings were treated with 10°C for 0, 24, 48, and 72 h, and then the leaves were collected for total RNA extraction and RT‐qPCR analysis. *HbeIF2* was used as an internal control. Data are means ± SD (*n* = 3). Bars with different letters indicate significant differences at *P* < 0.05, determined using anova with Tukey's multiple comparison test. (G, H) GUS‐staining images (G) and relative GUS activities (H) of the 7‐day‐old *HbCAT2::GUS* Arabidopsis seedlings treated with normal temperature (22°C), cold (4°C), 1 mM H_2_O_2_, or 10 μM abscisic acid (ABA) for 12 h. Bar = 0.5 cm. (I) Catalase activities were determined in the rubber tree seedlings treated with 10°C for 0, 24, 48 and 72 h. Data are means ± SD (*n* = 3). Bars with different letters indicate significant differences at *P* < 0.05, determined using anova with a Tukey's multiple comparison test. (J) Subcellular localization of HbCAT2. The empty GFP or GFP‐HbCAT2 was co‐expressed with peroxisomal marker mCherry‐SKL in the *Nicotiana benthamiana* leaves, and both the GFP and mCherry fluorescence images were captured by confocal microscopy. Bar = 20 μm.

Catalases are important antioxidant enzymes that scavenge excess H_2_O_2_ in plants, especially under stress conditions, while whether and how catalase plays a role in cold tolerance in rubber trees remains unclear. For this purpose, 3‐amino‐1,2,4‐triazole (3‐AT), an inhibitor of catalase, was employed to treat the *H. brasiliensis* leaves under normal (22°C) and cold (10°C) temperature conditions. The water‐treated and 3‐AT‐treated *H. brasiliensis* leaves displayed no significant differences under normal temperature conditions (Figure 1A,B); however, following cold stress treatment, the leaves treated with 3‐AT showed more severe damage as evidenced by less chlorophyll content (Figure 1A,B). Consistently, 3,3‐diaminobenzidine (DAB) and nitroblue tetrazolium (NBT) staining, which are qualitative or semi‐quantitative assays for ROS detection, showed stronger staining signals in the 3‐AT‐treated *H. brasiliensis* leaves than those in the water‐treated leaves under cold conditions (Figure 1C), indicating elevated levels of ROS, such as H_2_O_2_ and O_2_^•−^. To further characterize oxidative damage, we measured malondialdehyde (MDA) content, a biomarker of membrane lipid peroxidation (Weber et al., 2004). Our results showed that cold stress significantly increased MDA levels in *H. brasiliensis* leaves, while 3‐AT treatment further promoted this increase in MDA content (Figure 1D), suggesting exacerbated oxidative damage. Consistently, catalase activity in *H. brasiliensis* leaves was significantly inhibited by 3‐AT under both normal and cold temperature conditions (Figure 1E). These data suggest that catalase may play a positive role in cold tolerance in rubber trees.

Considering that AtCAT2 (AT4G35090) is the most active isoform in Arabidopsis catalase family, we performed phylogenetic analysis and sequence alignment, revealing that the protein LOC110660980 in *H. brasiliensis* was homologous to the well‐studied AtCAT2 in Arabidopsis, and thus designated as HbCAT2 (Figure S1). HbCAT2 is highly conserved in higher plants, but shows closer similarity to a catalase protein found in the tropical crop *Manihot esculenta* (Figure S1). To examine how *HbCAT2* responds to cold, *H. brasiliensis* seedlings were exposed to normal and low temperatures, and their leaves were then subjected to RNA extraction and reverse transcriptase‐quantitative polymerase chain reaction (RT‐qPCR). Our results showed that *HbCAT2* transcription increased significantly with prolonged cold exposure (Figure 1F; Figure S2A). Consistently, HbCAT2 protein levels were increased in *H. brasiliensis* leaves following cold treatment (Figure S2B). To study the expression pattern of *HbCAT2* in plants, we cloned its native promoter and generated *HbCAT2::GUS* transgenic Arabidopsis plants, where *GUS* expression was driven by the *HbCAT2* promoter. Consistent with the RT‐qPCR results (Figure 1F), cold stress treatment significantly increased the GUS activity in the *HbCAT2::GUS* seedlings compared to untreated controls (Figure 1G,H). Additionally, H_2_O_2_ and ABA also strongly induced GUS activity in these seedlings (Figure 1G). Consistently, catalase activity was much higher in the cold‐treated *H. brasiliensis* seedlings than that in the untreated control (Figure 1I). These findings indicate that both *HbCAT2* transcription and catalase activity are induced by cold stress in *H. brasiliensis*. To investigate the subcellular localization of HbCAT2, GFP was fused to the N‐terminus of the HbCAT2 protein, creating GFP‐HbCAT2. Both GFP and GFP‐HbCAT2 were transiently co‐expressed with mCherry‐SKL, a peroxisome marker, in *Nicotiana benthamiana* (*N. benthamiana*) leaves. Free GFP was found in the cytosol and nucleus, but not in peroxisomes (Figure 1J). In contrast, GFP‐HbCAT2 was co‐localized with mCherry‐SKL (Figure 1J), indicating that HbCAT2 is a peroxisomal protein.

### HbCAT2 confers cold tolerance in *H. brasiliensis* and Arabidopsis

**Figure 2:**
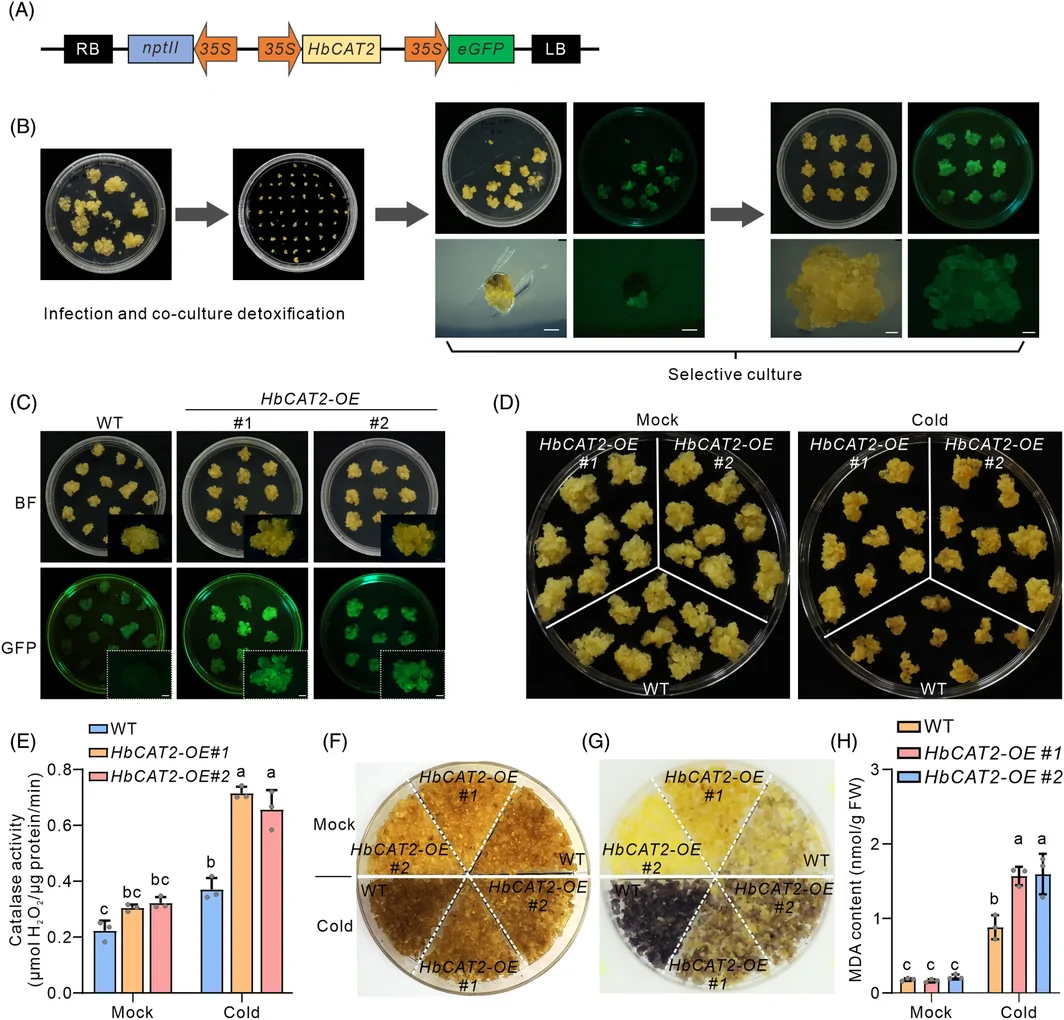
Overexpression of *HbCAT2* promotes cold tolerance in *Hevea brasiliensis*. (A) Schematic representation of *HbCAT2*‐overexpressing vectors. (B) A workflow of selecting paromomycin‐resistant transgenic callus lines. (C) Bright field (BF) and GFP fluorescence images of wild‐type and *HbCAT2‐OE* transgenic callus lines. Bars = 1 mm. (D) Growth and browning phenotypes of the wild‐type and *HbCAT2‐OE* calli treated with normal temperature (22°C) or cold (10°C) for 21 days. (E) Catalase activities in the wild‐type and *HbCAT2‐OE* calluses treated with normal temperature (22°C) or cold (10°C) for 7 days. Data are means ± SD (*n* = 3). Bars with different letters indicate significant differences at *P* < 0.05, determined using anova with a Tukey's multiple comparison test. (F, G) 3,3‐Diaminobenzidine (DAB) and nitroblue tetrazolium (NBT) staining images of the wild‐type and *HbCAT2‐OE* calli treated with normal temperature (22°C) or cold (10°C) for 1 h. (H) Malondialdehyde (MDA) content in the wild‐type and *HbCAT2‐OE* calli treated with 22°C or 10°C for 3 days. Data are means ± SD (*n* = 3). Bars with different letters indicate significant differences at *P* < 0.05, determined using anova with a Tukey's multiple comparison test.

To investigate whether HbCAT2 contributes to cold tolerance in *H. brasiliensis*, we generated transgenic *HbCAT2*‐overexpression (*HbCAT2‐OE*) *H. brasiliensis* calli (Figure 2A–C). Two independent transgenic callus lines (*HbCAT2‐OE* #1 and #2) with higher *HbCAT2* transcription and HbCAT2 protein levels (Figure S3) were subsequently analyzed for their cold tolerance. While *HbCAT2‐OE* calli grew similarly to the wild‐type under normal conditions, they exhibited markedly less growth inhibition and browning after prolonged cold stress (Figure 2D). These results suggest that overexpression of *HbCAT2* enhances cold tolerance in *H. brasiliensis* calli. In addition, catalase activity was significantly higher in the *HbCAT2‐OE* calli compared to the wild‐type under both normal and cold conditions (Figure 2E). Consistent with the phenotypic observations, DAB and NBT staining indicated similar H_2_O_2_ and O_2_^•−^ levels in both the wild‐type and *HbCAT2‐OE* calli under normal conditions, but lower levels in *HbCAT2‐OE* calli than in the wild‐type under cold conditions (Figure 2F,G). In addition, the MDA content was lower in the *HbCAT2‐OE* calli than in the wild‐type under cold conditions (Figure 2H). These results demonstrate that *HbCAT2* overexpression enhances cold tolerance in *H. brasiliensis* calli by increasing catalase activity and reducing ROS accumulation.

Moreover, cold tolerance assays were further carried out in transgenic Arabidopsis plants. We expressed *HbCAT2* in Arabidopsis (*35S::GFP‐HbCAT2*) (Figure S4A,B). Two transgenic lines with high *HbCAT2* expression levels and comparable growth phenotypes (*35S::GFP‐HbCAT2* #6 and #11) were selected for cold tolerance analysis (Figure S4C). As expected, the transgenic seedlings developed significantly longer primary roots and exhibited higher catalase activity than the wild‐type under cold stress (Figure S4D–F). Consistent with the function of CAT2 as a H_2_O_2_‐scavenging enzyme, DAB staining showed a clear reduction in H_2_O_2_ levels in these *35S::GFP‐HbCAT2* lines, while NBT staining revealed only a marginal decrease in superoxide accumulation (Figure S4G,H). This differential effect supports the specific role of CAT2 in regulating H_2_O_2_ homeostasis rather than general ROS scavenging. In addition, soil‐grown *35S::GFP‐HbCAT2* plants also displayed significantly higher survival rates after freezing treatment (Figure S4I,J). Together, these results suggest that HbCAT2 may enhance cold tolerance across species by attenuating excessive ROS accumulation through elevated catalase activity.

### HbCBF1 binds to and activates *HbCAT2* to increase plant cold tolerance

**Figure 3:**
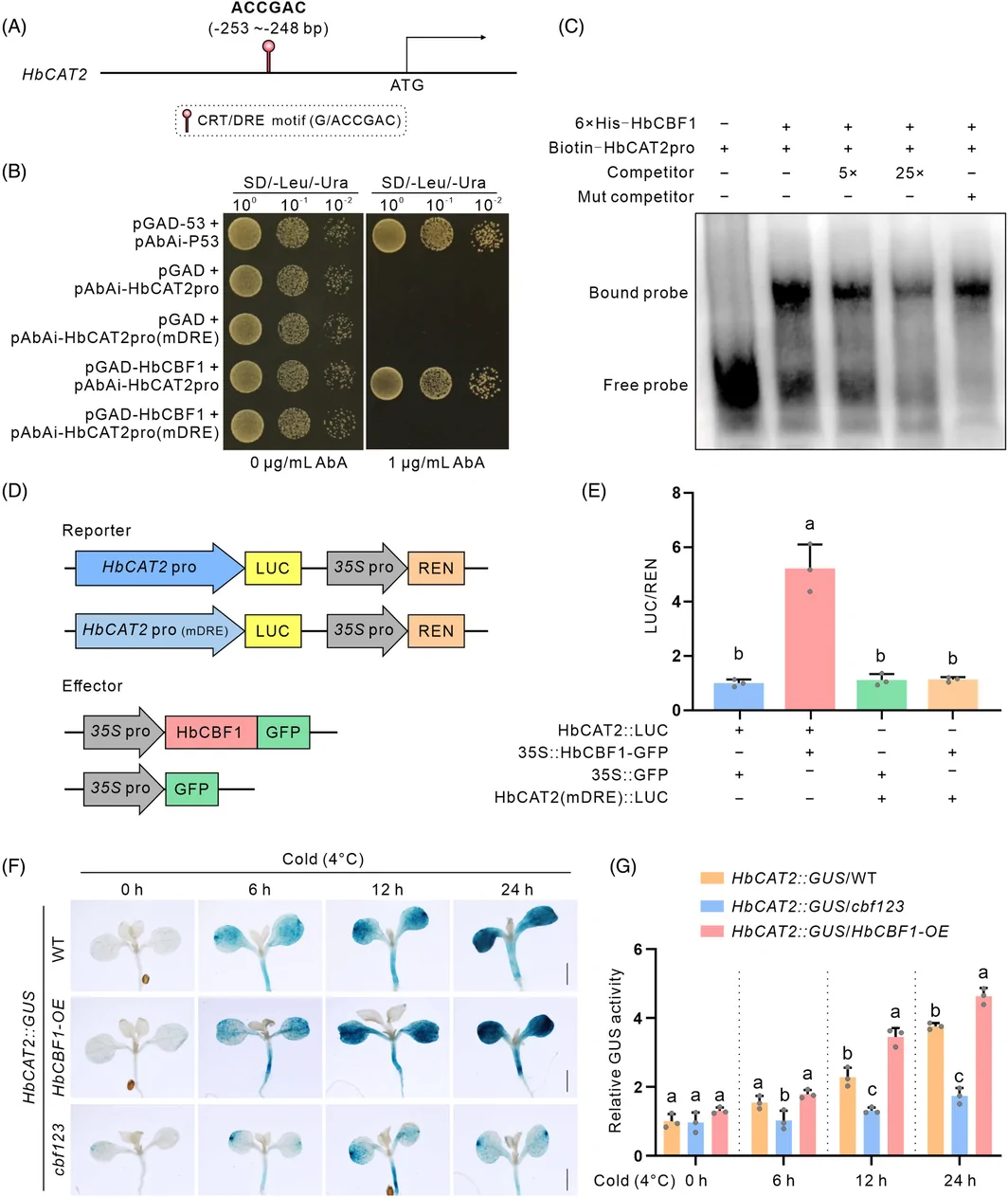
HbCBF1 binds to and activates *HbCAT2*. (A) A diagram showing a typical CRT/DRE motif in the *HbCAT2* promoter. (B) Yeast one‐hybrid experiment showing the binding of HbCBF1 to the *HbCAT2* promoter specifically at the DRE motif. The wild‐type and DRE‐mutated HbCAT2 promoters were respectively cloned into the pAbAi vector and then co‐transformed with the AD‐HbCBF1 into the Y1H Gold yeast cells. The transformed yeast cells were selected on SD/‐Leu‐Ura medium containing different concentrations of AbA. (C) EMSA assay showing the *in vitro* binding of HbCBF1 to *HbCAT2* promoter. Purified HbCBF1 protein was mixed with biotin‐labeled *HbCAT2* promoter (probe) and then subjected to a native gel. The non‐labeled and DRE motif‐mutated probes were used as the competitors. (D, E) A diagram (D) showing the reporters (HbCAT2::LUC and HbCAT2(mDRE)::LUC) and the effectors (35S::HbCBF1‐GFP and GFP), respectively. HbCAT2::LUC or HbCAT2(mDRE)::LUC was co‐expressed with 35S::HbCBF1‐GFP or GFP in the *Nicotiana benthamiana* leaves, and then the activities of LUC and REN were determined. The ratio of LUC to REN was calculated (E). Data are means ± SD (*n* = 3). Bars with different letters indicate significant differences at *P* < 0.05, determined using anova with a Tukey's multiple comparison test. (F, G) GUS‐staining images (F) of the 7‐day‐old seedlings of *HbCAT2::GUS, HbCAT2::GUS HbCBF1‐OE*, and *HbCAT2::GUS cbf123* Arabidopsis plants treated with cold for 0, 6, 12 or 24 h, and the GUS activities were measured (G). Data are means ± SD (*n* = 3). Bars with different letters indicate significant differences at *P* < 0.05, determined using anova with a Tukey's multiple comparison test. Bar = 0.5 cm.

To investigate how *HbCAT2* transcription is upregulated by cold in *H. brasiliensis*, the cis‐elements of the *HbCAT2* promoter were analyzed. A typical CRT/DRE motif (G/ACCGAC) was identified in the *HbCAT2* promoter (Figure 3A), suggesting that it could be targeted by CBFs, a subset of transcription factors that control cold‐responsive gene expression and cold tolerance (Sun et al., 2025). A previous study identified HbCBF1 as a functional CBF transcription factor that promotes cold tolerance in *H. brasiliensis* (Cheng et al., 2015). Therefore, we tested whether HbCBF1 binds to and activates *HbCAT2*. To achieve this, we cloned the coding sequence (CDS) of HbCBF1 along with a small fragment of the *HbCAT2* promoter that includes the motif ACCGAC, then performed a yeast one‐hybrid experiment. Our results showed that HbCBF1 could bind to the *HbCAT2* promoter, while mutation of the ACCGAC motif blocked their binding (Figure 3B), demonstrating that HbCBF1 targets the *HbCAT2* promoter by binding to the CRT/DRE motif. To confirm that HbCBF1 can directly bind to *HbCAT2* promoter, we performed EMSA assay by incubating purified 6 × His‐tagged HbCBF1 protein with biotin‐labeled *HbCAT2* promoter DNA as a hot probe. The results showed that HbCBF1 could bind to the *HbCAT2* promoter, while their binding was extensively competed by the non‐labeled probes but not by the mutated non‐labeled probes (Figure 3C). These results clearly revealed that HbCBF1 directly binds to the *HbCAT2* promoter *in vitro* and *in vivo*.

We next tested how HbCBF1 affects *HbCAT2* promoter activity by performing a dual‐luciferase (LUC) reporter gene assay where both *35S::HbCBF1‐GFP* as an effector and *HbCAT2::LUC* as a reporter were co‐expressed in *N. benthamiana* leaves (Figure 3D). The results showed that HbCBF1 significantly activated *HbCAT2::LUC* activity, whereas such activation was totally compromised when the CRT/DRE motif‐mutated *HbCAT2::LUC* was employed as a reporter (Figure 3E), indicating that HbCBF1 binds to the *HbCAT2* promoter to activate its expression in plants.

To confirm the role of HbCBF1 in regulating cold‐induced *HbCAT2* expression in plants, we created *35S::HbCBF1‐GFP* (*HbCBF1‐OE*) plants in Arabidopsis and crossed them with *HbCAT2::GUS* plants, resulting in *HbCAT2::GUS HbCBF1‐OE* plants. The seedlings were then subjected to cold treatment, and the GUS staining revealed that cold strongly induced GUS activity in the *HbCAT2::GUS* seedlings over time, with even stronger induction in the cold‐treated *HbCAT2::GUS HbCBF1‐OE* plants (Figure 3F,G), indicating that HbCBF1 can activate *HbCAT2* expression in plants. Additionally, we crossed *HbCAT2::GUS* plants with the Arabidopsis *cbf123* triple mutant that exhibits extreme sensitivity to cold stress (Jia et al., 2016), producing *HbCAT2::GUS cbf123* plants. Both *HbCAT2::GUS* and *HbCAT2::GUS cbf123* seedlings were exposed to normal and cold temperatures. Following GUS staining, we found that cold‐induced GUS activity in *HbCAT2::GUS* plants was greatly compromised in *HbCAT2::GUS cbf123* plants (Figure 3F,G). These findings suggest that CBF transcription factors in both *A. thaliana* and *H. brasiliensis* are able to transcriptionally activate *HbCAT2* expression in plant response to cold.

### HbCBF1 may function upstream of *HbCAT2* in plant cold tolerance

Our above results highlighted a role of HbCBF1 in cold‐induced *HbCAT2* expression, leading us to investigate its regulatory impact on plant cold tolerance. If HbCBF1 enhances plant cold tolerance by promoting catalase expression and activity, disrupting catalase activity may reduce the cold tolerance of *HbCBF1*‐overexpressing plants. We first carried out a dual‐LUC reporter gene assay to test whether HbCBF1 affects the *AtCAT2* promoter activity in plants. Our results showed that the LUC expression driven by the *AtCAT2* promoter was significantly activated by HbCBF1 (Figure S5), suggesting that HbCBF1 transcriptionally activates the expression of both *AtCAT2* and *HbCAT2*. Then, we analyzed whether HbCBF1 confers cold tolerance partially through catalase activity in Arabidopsis plants. We generated *HbCBF1‐OE cat2‐1* plants through genetic crossing of *HbCBF1‐OE* with the Arabidopsis *cat2‐1* mutant. We then treated the wild‐type, *HbCBF1‐OE*, *HbCBF1‐OE cat2‐1*, and *cat2‐1* seedlings with cold. Under normal conditions, all plants had similar root lengths (Figure S6A,B). However, under cold conditions, *HbCBF1‐OE* plants had longer roots than the wild‐type, while *HbCBF1‐OE cat2‐1* plants had significantly shorter roots than *HbCBF1‐OE* plants (Figure S6A,B), suggesting that catalase activity is required for HbCBF1‐mediated cold tolerance in Arabidopsis. Similar findings were observed in soil‐grown plants, where *HbCBF1‐OE* plants had a higher survival rate compared to *HbCBF1‐OE cat2‐1* plants (Figure S6C,D). Consistently, cold‐induced catalase activity was lower in *HbCBF1‐OE cat2‐1* plants than in *HbCBF1‐OE* plants (Figure S6E), while cold‐induced ROS accumulation, visualized by DAB and NBT staining, was higher in *HbCBF1‐OE cat2‐1* plants than in *HbCBF1‐OE* plants (Figure S6F,G).

Next, we investigated whether enhanced catalase activity by *HbCAT2* overexpression could rescue cold hypersensitivity of the Arabidopsis *cbf123* mutant. To this end, we crossed *HbCAT2‐OE* plants with *cbf123*, generating *HbCAT2‐OE cbf123* plants, and exposed their seedlings to cold temperatures. Our findings revealed that the *cbf123* mutant was highly sensitive to cold stress, while *HbCAT2‐OE* partially mitigated this sensitivity in terms of root elongation in MS medium (Figure S7A,B) and survival rate in soil (Figure S7C,D). Additionally, catalase activity was increased and ROS accumulation was reduced in *HbCAT2‐OE cbf123* plants compared to those in the *cbf123* mutant plants under cold stress (Figure S7E,F). These results demonstrate that increasing *HbCAT2* expression and catalase activity can partially enhance the cold tolerance of the *cbf123* mutant, suggesting that CBF transcription factors may function upstream of *HbCAT2* in improving plant cold tolerance.

### HbSnRK2.6A interacts with and phosphorylates HbCAT2 to promote catalase activity

**Figure 4:**
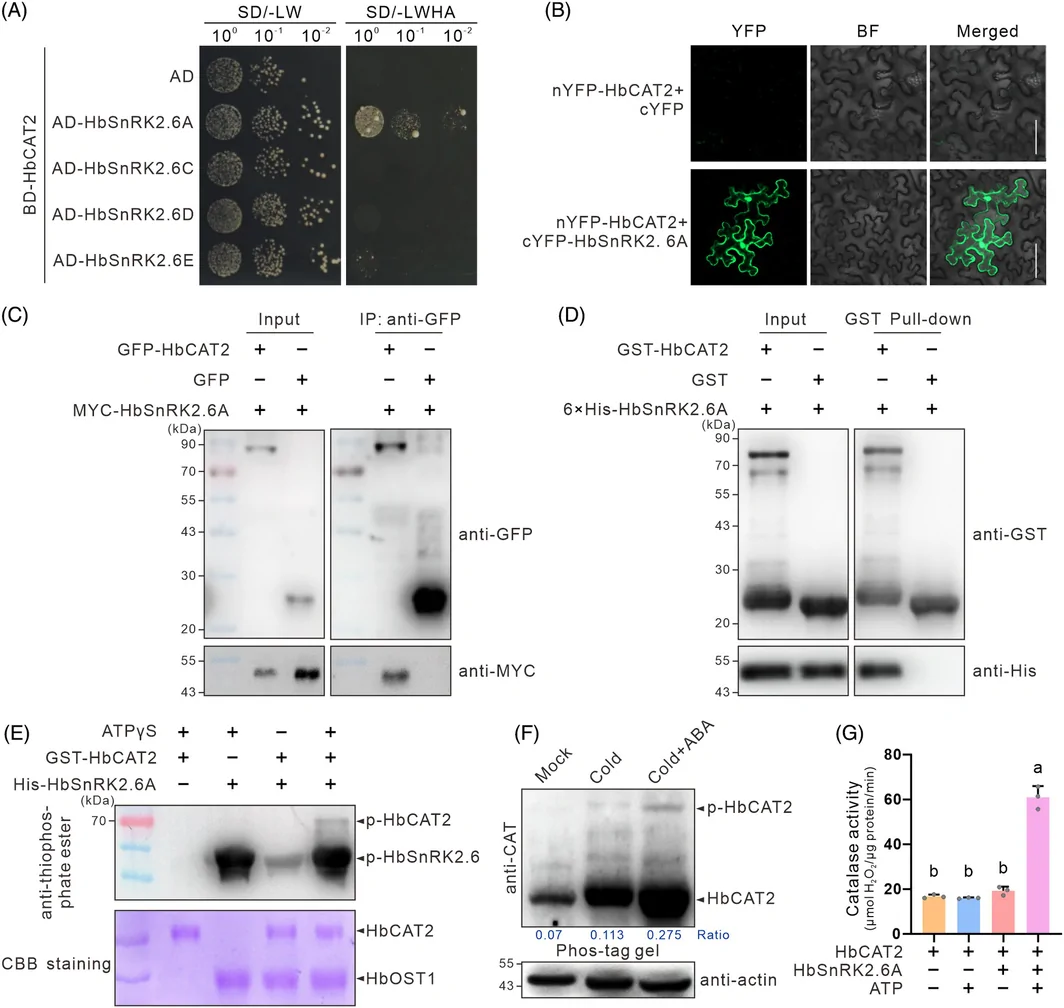
HbSnRK2.6 interacts with and phosphorylates HbCAT2. (A) Yeast two‐hybrid assay showing the interaction of HbCAT2 with HbSnRK2.6A. (B) The interaction between HbCAT2 and HbSnRK2.6A was assayed by bimolecular fluorescence complementation (BiFC). The nYFP‐HbCAT2 was co‐expressed with cYFP or cYFP‐HbSnRK2.6A in the *Nicotiana benthamiana* leaves, and the fluorescence images were captured under confocal microscopy. Bar = 50 μm. (C) Co‐immunoprecipitation (Co‐IP) experiment showing the interaction between HbCAT2 and HbSnRK2.6A. GFP or GFP‐tagged HbCAT2 was co‐expressed with MYC‐tagged HbSnRK2.6A in *N. benthamiana* leaves. Total proteins were extracted and immunoprecipitated with anti‐GFP antibody, and then the precipitates were determined with anti‐GFP and anti‐MYC antibodies, respectively. (D) The *in vitro* interaction between HbCAT2 and HbSnRK2.6A was determined by GST pull‐down. The purified GST or GST‐tagged HbCAT2 was incubated with 6 × His‐tagged HbSnRK2.6A, and the mixture was pulled down by GST beads. The eluted proteins were detected with anti‐GST and anti‐His antibodies, respectively. (E) *In vitro* phosphorylation of HbCAT2 mediated by HbSnRK2.6A. The purified GST‐HbCAT2 and His‐HbSnRK2.6A proteins were mixed at room temperature for 30 min in the presence or absence of ATPγS. The protein mixture was alkylated and then separated by SDS‐PAGE gel. The phosphorylation signals were detected by the anti‐thiophosphate ester antibody. (F) Phos‐tag gel showing increased phosphorylation of HbCAT2 in *Hevea brasiliensis* treated with cold and abscisic acid (ABA). Total proteins extracted from the rubber tree seedlings treated with normal temperature (22°C), cold (10°C), or cold + 10 μM ABA for 24 h were separated by SDS‐PAGE gel and then detected by anti‐CAT2 antibody. Actin was used as a loading control. The ratios of the phosphorylated HbCAT2 to unphosphorylated HbCAT2 are shown in blue. (G) HbSnRK2.6A increases the catalase activity of HbCAT2. The purified GST‐HbCAT2 was incubated with or without His‐HbSnRK2.6A or ATP at room temperature for 30 min, and then the catalase activities were measured. Data are means ± SD (*n* = 3). Bars with different letters indicate significant differences at *P* < 0.05, determined using anova with Tukey's multiple comparison test.

We noticed that catalase activity in cold‐treated *35S::GFP‐HbCAT2* plants was higher than in untreated plants, leading us to investigate how HbCAT2 activity is regulated in addition to transcriptional regulation in *H. brasiliensis*. Using HbCAT2 as the bait protein, we conducted yeast two‐hybrid (Y2H) screening assays against a cDNA library constructed from *H. brasiliensis* tissues. We identified HbSnRK2.6A, a protein kinase involved in plant cold tolerance, as an HbCAT2‐interacting protein (Figure 4A). Other members of the HbSnRK2.6A subfamily did not interact with HbCAT2 in yeast cells (Figure 4A). To confirm the interaction between HbSnRK2.6A and HbCAT2, we conducted an *in vivo* bimolecular fluorescence complementation (BiFC) assay in *N. benthamiana* leaves, co‐expressing HbCAT2 fused to the N‐terminal half of yellow fluorescent protein (nYFP) and HbSnRK2.6A fused to the C‐terminal half of YFP (cYFP). Strong YFP fluorescence was observed in leaves co‐expressing nYFP‐HbCAT2 and cYFP‐HbSnRK2.6A, but not in the negative controls (Figure 4B). The interaction was further confirmed by a co‐immunoprecipitation (Co‐IP) experiment, where MYC‐tagged HbSnRK2.6A and GFP‐tagged HbCAT2 were co‐expressed in *N. benthamiana* leaves and immunoprecipitated using an anti‐GFP antibody. The Co‐IP results showed that MYC‐HbSnRK2.6A was specifically detected by the anti‐MYC antibody among the proteins immunoprecipitated by the anti‐GFP antibody (Figure 4C), further confirming the *in vivo* interaction of HbCAT2 with HbSnRK2.6A. An *in vitro* GST pull‐down assay also demonstrated that 6 × His‐HbSnRK2.6A could be pulled down by GST‐HbCAT2 but not by empty GST protein (Figure 4D), indicating a physical interaction between HbCAT2 and HbSnRK2.6A. In addition, we examined whether HbSnRK2.6A interacts with Arabidopsis AtCAT2. Our Y2H and GST pull‐down experiments showed that HbSnRK2.6A directly interacts with AtCAT2 (Figure S8), suggesting conserved mechanism underlying SnRK2.6 in the regulation of catalase proteins in both *H. brasiliensis* and Arabidopsis.

We hypothesized that HbSnRK2.6A, as a protein kinase, may phosphorylate HbCAT2 through its interaction. To test this possibility, we expressed and purified GST‐tagged HbCAT2 and His‐tagged HbSnRK2.6A proteins in *Escherichia coli* and conducted *in vitro* kinase assay. Notably, HbCAT2 phosphorylation was observed only in the presence of both HbSnRK2.6A and ATP, but not in the absence of either HbSnRK2.6A or ATP (Figure 4E), indicating that HbSnRK2.6A specifically phosphorylates HbCAT2 *in vitro*. To detect HbCAT2 phosphorylation in rubber trees, total proteins extracted from *H. brasiliensis* seedlings treated with cold and ABA were analyzed using phos‐tag gel. Phosphorylation bands above the HbCAT2 protein were observed in cold‐ and ABA‐treated seedlings, but not in the untreated control (Figure 4F; Figure S2B), indicating that cold induces HbCAT2 protein phosphorylation in *H. brasiliensis*. To study whether HbSnRK2.6A affects the catalase activity of HbCAT2, we measured catalase activity of the purified HbCAT2 proteins in the presence and absence of HbSnRK2.6A or ATP. Our results showed that HbCAT2 activity was only greatly stimulated in the presence of both HbSnRK2.6A and ATP, while its activity remained unchanged when HbSnRK2.6A or ATP was lacking (Figure 4G), demonstrating that HbSnRK2.6A promotes HbCAT2 catalase activity through interaction and phosphorylation.

Using the PhosphoPredict algorithm (Song et al., 2017), four potential phosphorylation sites were identified on the HbCAT2 protein, including Ser‐9, Thr‐105, Ser‐347, and Ser‐437 (Figure S9A). Given that Ser‐9 and Thr‐105 are located in the N‐terminal region of HbCAT2, while Ser‐347 and Ser‐437 reside in the C‐terminal region (Figure S9A), we divided HbCAT2 into N‐terminal (residues 1–247) and C‐terminal (residues 248–492) fragments (Figure S9A). Y2H assays revealed that HbSnRK2.6A interacts with the C‐terminal fragment but not the N‐terminal fragment of HbCAT2 (Figure S9B). To validate the phosphorylation sites, we expressed and purified wild‐type HbCAT2 and five mutant variants, including HbCAT2‐S9A, HbCAT2‐T105A, HbCAT2‐S347A, HbCAT2‐S437A, and HbCAT2‐S347A/S437A. *In vitro* phosphorylation assays showed robust phosphorylation signals mediated by HbSnRK2.6A on wild‐type HbCAT2, HbCAT2‐S9A, and HbCAT2‐T105A, but no detectable signals on HbCAT2‐S347A, HbCAT2‐S437A, or HbCAT2‐S347A/S437A (Figure S9C). These results support the idea that Ser‐347 and Ser‐437 are required for HbSnRK2.6A‐dependent phosphorylation of HbCAT2 *in vitro*. To investigate how phosphorylation affects catalase activity, we measured the enzymatic activity of wild‐type and mutant HbCAT2 proteins. Consistent with the phosphorylation data, HbCAT2‐S9A and HbCAT2‐T105A exhibited catalase activity comparable to wild‐type HbCAT2, whereas HbCAT2‐S347A, HbCAT2‐S437A, and HbCAT2‐S347A/S437A showed significantly reduced catalase activity (Figure S9D). These findings confirm that Ser‐347 and Ser‐437 are critical residues for HbCAT2 enzymatic activity.

### ABA and HbSnRK2.6A are associated with increased catalase activity during cold stress

**Figure 5:**
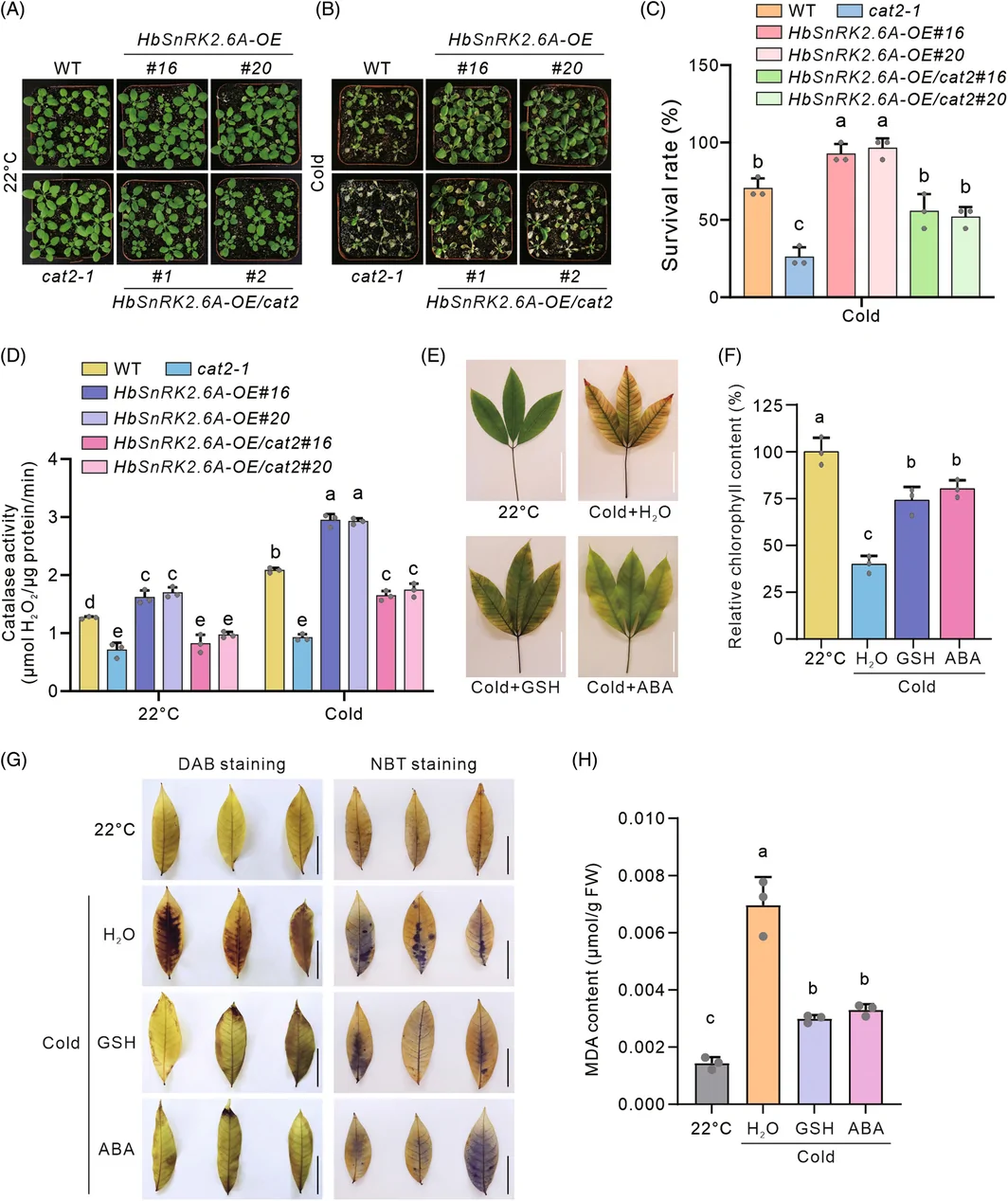
HbSnRK2.6A‐conferred cold tolerance is partly dependent on catalase. (A–C) Phenotype (A, B) and survival rate (C) of the wild‐type, *cat2‐1*, *HbSnRK2.6A‐OE* and *HbSnRK2.6A/cat2‐1* plants treated with freezing stress. 3‐week‐old wild‐type, *cat2‐1*, *HbSnRK2.6A‐OE* and *HbSnRK2.6A/cat2‐1* plants grown in soil were cold acclimated at 4°C for 2 days, then placed into a freezing chamber at 0°C, with the temperature dropping by 1°C per hour until −8°C. The seedlings were kept at 4°C for 12 h in the dark and grown for an additional 3 days at 22°C. Data are means ± SD (*n* = 3). Bars with different letters indicate significant differences at *P* < 0.05, determined using anova with Tukey's multiple comparison test. (D) Catalase activities were determined in (A). Data are means ± SD (*n* = 3). Bars with different letters indicate significant differences at *P* < 0.05, determined using anova with Tukey's multiple comparison test. (E, F) Leaf phenotype (E) of the *Hevea brasiliensis* seedling leaves treated with cold in the presence of H_2_O, GSH or abscisic acid (ABA). The *H. brasiliensis* seedlings were treated with H_2_O, 0.25 mM GSH or 10 μM ABA under 22°C or 10°C for 7 days, and then the chlorophyll content (F) was measured. Data are means ± SD (*n* = 3). Bars with different letters indicate significant differences at *P* < 0.05, determined using anova with Tukey's multiple comparison test. Bars = 5 cm. (G) 3,3‐Diaminobenzidine (DAB) and nitroblue tetrazolium (NBT) staining images of the *H. brasiliensis* seedling leaves shown in (E). Bars = 5 cm. (H) Malondialdehyde (MDA) content in the *H. brasiliensis* seedling leaves treated with H_2_O, 0.25 mM GSH or 10 μM ABA under 10°C conditions for 3 days. Data are means ± SD (*n* = 3). Bars with different letters indicate significant differences at *P* < 0.05, determined using anova with Tukey's multiple comparison test.

To investigate the genetic relationship between HbSnRK2.6A and HbCAT2 with regard to plant cold tolerance, we crossed the *HbSnRK2.6A‐OE* plants (Wang et al., 2021) with the Arabidopsis *cat2‐1* mutant, resulting in *HbSnRK2.6A‐OE cat2‐1* plants, and tested its cold sensitivity. The seedlings of the wild‐type, *cat2‐1*, *HbSnRK2.6A‐OE*, and *HbSnRK2.6A‐OE cat2‐1* plants were subjected to freezing stress, and their survival rate was determined. Our results showed that overexpression of *HbSnRK2.6A* extensively increased the survival rate in the wild‐type plants, but only slightly in the *cat2‐1* mutant plants (Figure 5A–C), supporting that CAT2 functions necessarily in HbSnRK2.6A‐mediated cold tolerance. In line with the phenotypes, catalase activity was lower in the *HbSnRK2.6A‐OE cat2‐1* plants than in the *HbSnRK2.6A‐OE* plants under cold conditions (Figure 5D). These findings collectively suggest that HbSnRK2.6A enhances cold tolerance at least partially through activating HbCAT2 activity in plants.

Given that HbSnRK2.6A can be activated by the phytohormone ABA and HbSnRK2.6A enhances cold tolerance partly through activating catalase activity in Arabidopsis, we speculate that scavenging excess H_2_O_2_ may enhance cold tolerance in *H. brasiliensis*. For this purpose, GSH, a H_2_O_2_‐scavenger, was sprayed onto the cold‐treated *H. brasiliensis* leaves. Our results obviously showed that cold stress severely impacted rubber tree growth as evidenced by reduced chlorophyll content, whereas GSH significantly improved cold tolerance of the rubber trees (Figure 5E,F). Consistent with the cold phenotype, cold‐induced accumulation of ROS and MDA was extensively suppressed by GSH treatment in *H. brasiliensis* leaves (Figure 5G,H). Intriguingly, exogenous ABA treatment also remarkably repressed cold‐induced accumulation of ROS and MDA and promoted cold tolerance in *H. brasiliensis*, suggesting a correlation between ABA and ROS homeostasis in the cold response.

## DISCUSSION

**Figure 6:**
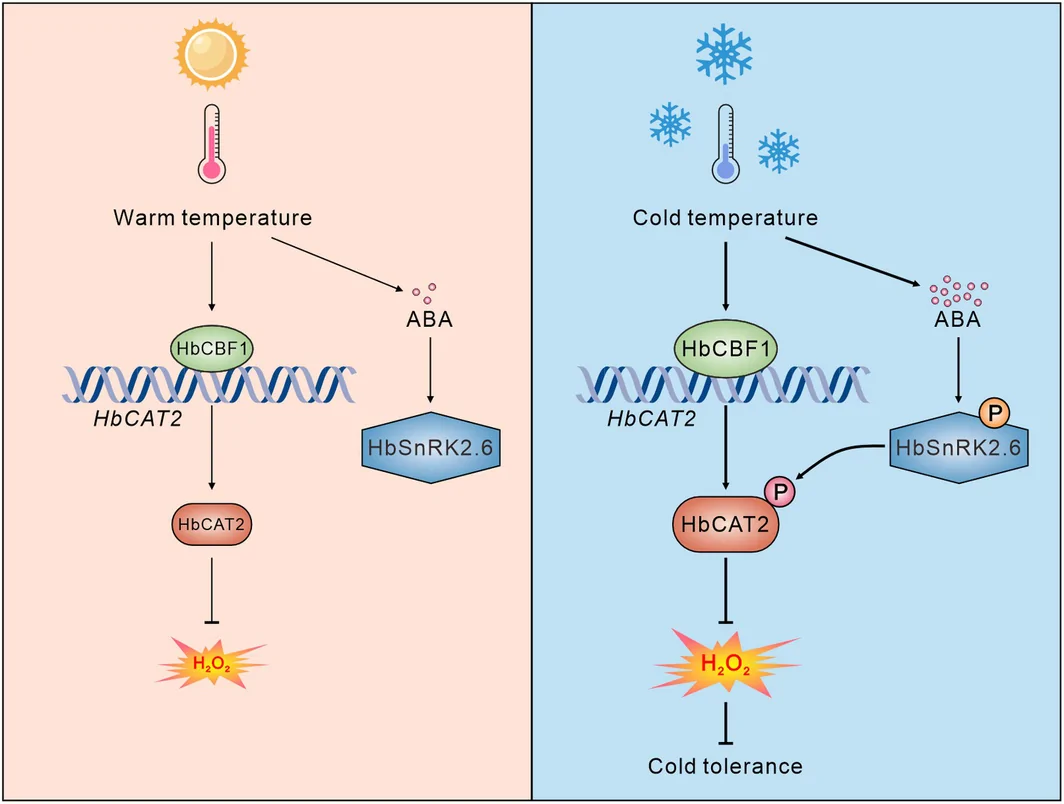
A working model showing HbCBF1/HbSnRK2.6‐HbCAT2 regulatory module in cold tolerance in *Hevea brasiliensis*. Under warm temperature conditions, HbCAT2 expression and activity are low, maintaining basal H_2_O_2_ levels in *H. brasiliensis*. Under cold temperature conditions, HbCBF1 transcription factor binds to the *HbCAT2* promoter to upregulate its expression; meanwhile, abscisic acid (ABA) activates protein kinase SnRK2.6A to phosphorylate HbCAT2, promoting its catalase activity. Both mechanisms contribute to increased catalase activity and decreased H_2_O_2_ accumulation, thereby enhancing cold tolerance in *H. brasiliensis*.

Cold stress is a critical environmental factor affecting the geographical distribution, growth, and productivity of crops, leading to significant economic losses (Chinnusamy et al., 2007; Kidokoro et al., 2022; Shi et al., 2018). Overaccumulation of ROS causes oxidative damage and impairs cold tolerance in *H. brasiliensis*, while how ROS are balanced and regulated in the cold response of *H. brasiliensis* remains largely unclear. Here, we identified HbCAT2, a positive factor regulating cold tolerance in *H. brasiliensis* and Arabidopsis, and demonstrate that both HbCBF1‐mediated transcriptional activation and HbSnRK2.6A‐mediated phosphorylation may contribute to the ROS‐scavenging ability of HbCAT2 (Figure 6).

*HbCAT2* is upregulated by cold and H_2_O_2_ (Figure 1F–H), suggesting its role in plant responses to low temperature and oxidative stress. This is supported by the fact that overexpression of *HbCAT2* obviously reduced the ROS levels in the wild‐type *H. brasiliensis* calli under cold conditions (Figure 2F–H). Similar to Arabidopsis, HbCAT2 is mainly localized in peroxisomes (Figure 1J), which is consistent with its H_2_O_2_‐scavenging role in this organelle. While the interaction between HbSnRK2.6A and HbCAT2 occurs mainly in the cytoplasm, with partial localization at the plasma membrane and in the nucleus (Figure 4B), suggesting that HbSnRK2.6A phosphorylates HbCAT2 and promotes its activity before its entry into peroxisomes. The observation that HbSnRK2.6A interacts with HbCAT2 mainly in the cytoplasm, rather than in peroxisomes where CAT2 primarily functions, aligns with emerging evidence that critical regulatory modifications of catalases often occur in the cytoplasm prior to their translocation to peroxisomes. For example, the molecular chaperone NO CATALASE ACTIVITY 1 (NCA1) interacts with CAT2 in the cytoplasm to facilitate its proper folding and assembly before targeting to peroxisomes in Arabidopsis (Li et al., 2015). Similarly, the kinase CALMODULIN‐BINDING RECEPTOR‐LIKE CYTOPLASMIC KINASE 3 (CRCK3) phosphorylates CAT2 in the cytoplasm, which is essential for enhancing CAT2 activity and promoting salt tolerance in Arabidopsis (Zhuang et al., 2024). These studies collectively support our finding that the cytoplasmic interaction between HbSnRK2.6A and HbCAT2 represents a critical regulatory step, where HbSnRK2.6A‐mediated phosphorylation may modulate HbCAT2 activity before it reaches its final functional destination.

Notably, the Arabidopsis SnRK2.6 has five homologs in *H. brasiliensis*, SnRK2.6A, SnRK2.6B, SnRK2.6C, SnRK2.6D, and SnRK2.6E, while only SnRK2.6A showed strong interaction with HbCAT2 (Figure 4A), suggesting that the number of SnRK2.6 genes in rubber trees has been expanded with diversified interacting partners and functions. SnRK2.6, also termed Open Stomata 1 (OST1), is a Ser/Thr protein kinase phosphorylating downstream ABA‐responsive transcription factors and ion channels, which is essential for ABA response and multiple stress tolerance in various plant species (Zhang et al., 2025). In a previous study, OST1 was demonstrated to interact with and phosphorylate ICE1 to enhance its protein stability, thus improving cold tolerance in Arabidopsis (Ding et al., 2015). Consistent with this, we previously also found that HbSnRK2.6 plays an important role in cold tolerance in *H. brasiliensis* (Wang et al., 2021). In this study, we further demonstrate that HbSnRK2.6A from *H. brasiliensis* can interact with and phosphorylate HbCAT2, leading to higher catalase activity and lower ROS accumulation in plant response to cold stress (Figure 4). HbSnRK2.6A interacts with AtCAT2 (Figure S8), and overexpression of *HbSnRK2.6A* enhances cold tolerance in Arabidopsis (Figure 5A–C), suggesting a conserved mechanism of SnRK2.6 in the regulation of cold tolerance in *H. brasiliensis* and Arabidopsis. We infer that SnRK2.6 in Arabidopsis probably has a similar function in cold tolerance by regulating ROS levels, as the higher survival rate of *HbSnRK2.6A‐OE* plants was greatly impaired by disrupting Arabidopsis CAT2 under cold conditions (Figure 5A–C).

In this study, we provide evidence to show that HbCBF1 directly binds to the DRE/CRT motif within *HbCAT2* promoter, and HbCBF1‐mediated activation of *HbCAT2* expression is dependent on this motif (Figure 3A–E). Although the Arabidopsis *AtCAT2* promoter has no typical CBF1‐recognizing DRE/CRT motif, our results demonstrate that HbCBF1 could significantly activate *AtCAT2* promoter activity (Figure S5). This suggests that CBF transcription factors bind to atypical DRE/CRT motifs to directly activate its expression, or indirectly activate its expression by collaborating with other transcription factors that bind to the *AtCAT2* promoter. This hypothesis is supported by two sets of results: (1) the Arabidopsis *cbf123* mutant accumulated higher ROS levels than wild‐type plants under cold conditions (Figure S7F); and (2) the increased cold tolerance of *HbCBF1‐OE* plants was compromised by the *cat2‐1* mutation in Arabidopsis (Figure S6A–D).

The *HbCAT2::GUS* reporter could be significantly stimulated by cold stress in the Arabidopsis wild‐type plants but not in the *cbf123* mutants (Figure 3F,G), suggesting that *HbCAT2* promoter can be bound and activated by Arabidopsis CBF transcription factors. We speculate that the CBF transcription factors are very conserved between Arabidopsis and *H. brasiliensis* in terms of their DNA‐binding and activation activities. Our results suggest that catalase activity is required for plant cold tolerance as higher cold tolerance of the *HbCBF1‐OE* plants was partially compromised by the *cat2‐1* mutation in Arabidopsis (Figure S6A–D), while *HbCAT2* overexpression could partially restore the cold hypersensitivity of the Arabidopsis *cbf123* mutant (Figure S7A–D). These results obtained from *H. brasiliensis* and Arabidopsis suggest a critical role of catalase in cold tolerance through modulating ROS homeostasis in plants.

While our results in both *H. brasiliensis* and Arabidopsis provide insights into the conserved function of CAT2 in plant cold tolerance, it is important to acknowledge the biological differences between Arabidopsis and *H. brasiliensis*. Arabidopsis is a temperate annual herb that has evolved sophisticated mechanisms to tolerate freezing temperatures, whereas *H. brasiliensis* is a tropical perennial tree that is highly sensitive to even mild chilling stress. In this study, we found that exposing Arabidopsis to freezing temperature (−8°C) while *H. brasiliensis* to non‐freezing temperature (only 10°C) causes severe damages, indicating that *H. brasiliensis* is highly sensitive to low temperatures. Therefore, it is of great importance to identify the genes involved in *H. brasiliensis* cold tolerance and investigate the underlying mechanisms. In this study, we have tested the role of HbCAT2 in cold tolerance not only in the model plant *A. thaliana* but also in *H. brasiliensis* using transgenic *HbCAT2‐OE* calli, revealing that HbCAT2 is a positive regulator of plant cold tolerance. Pharmacological experiments demonstrate that inhibiting catalase activity by 3‐AT attenuated cold tolerance while scavenging of excess H_2_O_2_ by exogenously applied GSH could elevate cold tolerance in *H. brasiliensis* (Figure 5E–H). While these results are promising, it is important to note that further field trials would be required to evaluate the potential of exogenous GSH application as a method for promoting cold stress tolerance in agricultural production of rubber trees.

## MATERIALS AND METHODS

### Plant materials and treatment

Seedlings of the *H. brasiliensis* cultivar Reyan 7‐33‐97 used in this study were purchased from Chinese Academy of Tropical Agricultural Sciences and cultivated in the greenhouse of Hainan University, Hainan Province, China. To determine the expression of *HbCAT2* in response to cold, the *H. brasiliensis* seedlings were exposed to 10°C for the indicated time. For the GSH and ABA treatment, water, 5 mM 3‐AT, 0.25 mM GSH or 10 μM ABA solution was sprayed on the leaves of the rubber tree seedlings, and then the treated seedlings were transferred to a growth chamber at 10°C under a 16 h light/8 h dark cycles with 75% humidity for 7 days.

*Arabidopsis thaliana* Col‐0 wild‐type plants were used in this study. The seeds of the *cat2‐1* (Salk_076998) mutant, *cbf123* triple mutant, and *HbSnRK2.6A‐OE* transgenic plants were previously reported (Jia et al., 2016; Yuan, Liu, & Lu, 2017; Zeng et al., 2025). Seeds were surface sterilized for 5 min in 5% commercial bleach, washed three times with sterile water, and plated on Murashige and Skoog (MS) medium (pH 5.8) containing 0.8% agar. Plants were stratified at 4°C for 3 days in the dark, and then transferred to chambers. The seedlings were grown vertically at 22°C and 100 μmol m^−2^ sec^−1^ illumination under 16 h light/8 h dark conditions.

The freezing tolerance test was performed according to previous reports (Ding et al., 2015; Liu et al., 2022). Briefly, 3‐week‐old wild‐type, mutant, and transgenic plants grown in soil were cold acclimated at 4°C for 2 days, then placed into a freezing chamber at 0°C, with the temperature dropping by 1°C per hour until −8°C. The plants were kept at 4°C for 12 h in the dark and grown for an additional 3 days at 22°C under a 16 h light/8 h dark cycle.

### RT‐qPCR

The untreated and treated rubber tree seedling leaves or Arabidopsis plant seedlings were harvested for total RNA isolation. The total RNA was extracted using RNAkey Reagent (Seven Biotech, Beijing, China; Cat#: SM129‐02) according to the instruction manual. The first‐strand cDNA synthesis and RT‐PCR were performed according to the manufacturer's instructions (Yugong Biotech, Lianyungang, China; Cat#: EG15133S). The constitutively expressed genes *Actin2/8* for Arabidopsis and *HbeIF2* for *H. brasiliensis* were used as internal controls, respectively. The primer sequences are listed in Table S1.

### Dual‐LUC reporter gene assay

To evaluate the role of HbCBF1 in regulating *HbCAT2* transcription, the full‐length CDS of *HbCBF1* was cloned into the pEGAD vector and the 1865‐bp genomic DNA sequence upstream of the *HbCAT2* translation start site (ATG) was cloned into the pGreenII0800‐LUC vector, resulting in 35S::GFP‐HbCBF1 and HbCAT2::LUC plasmid constructs, respectively. The dual‐LUC reporter gene assay was carried out according to our previously reported method (Lu et al., 2025). Briefly, the HbCAT2::LUC was used as a reporter, and the Renilla luciferase (REN) under the control of the *35S* promoter in the pGreenII0800‐LUC vector was used as an internal control. The 35S::GFP‐HbCBF1 was used as an effector. HbCAT2::LUC was transiently co‐expressed with GFP or 35S::GFP‐HbCBF1 in the *N. benthamiana* leaves via *Agrobacterium*‐mediated transformation for 3 days in the dark. Then, the leaves were collected and the activities of LUC and REN were measured. The primers used for the construction of these plasmids are listed in Table S1.

### EMSA assay

EMSA was performed according to our previous reports (Lu et al., 2025). Briefly, the full‐length CDS of *HbCBF1* was cloned into the pET28a vector at the *EcoR*I site. The resulting pET28a‐HbCBF1 plasmid was transformed and induced in the *E. coli* BL21 (DE3) strain, and then the 6 × His‐tagged HbCBF1 protein was purified by NTA‐beads. The EMSA assay was performed using an EMSA/Gel‐Shift kit (Beyotime, Shanghai, China; Cat#: GS002), according to the manufacturer's instructions. Briefly, biotin‐labeled probes were incubated with or without purified 6 × His‐HbCBF1 in the EMSA/Gel‐Shift Binding Buffer (Beyotime, Cat#: GS005) for 30 min at room temperature. For the EMSA competition experiments, non‐labeled non‐mutated and mutated probes used as competitors were added to the binding reactions, respectively. The probe sequences are listed in Table S1.

### GUS staining and GUS activity

The untreated and cold‐treated *HbCAT2::GUS* transgenic plant seedlings were incubated in the GUS‐staining buffer (100 mM sodium phosphate buffer, pH 7.5, 10 mM EDTA, 0.5 mM potassium ferricyanide, 0.5 mM potassium ferrocyanide, 1 mM 5‐bromochloro‐3‐indolyl‐b‐d‐glucuronide, and 0.1% Triton X‐100). The samples were then rinsed with 75% ethanol several times to remove the chlorophyll, and the images were then taken. The GUS activities were assayed according to a method described previously (Lu et al., 2023).

### Yeast one‐hybrid

The full‐length CDS of *HbCBF1* and 250‐bp promoter sequence upstream of the *HbCAT2* translation start site (ATG) were cloned into the pGADT7 and pAbAi vectors, respectively. Yeast transformants were selected on double dropout medium lacking Leu and Ura (SD/‐LU) and the protein‐promoter interaction was determined on SD/‐LU medium containing 1 μg ml^−1^ AbA. Primer sequences are listed in Table S1.

### Yeast two‐hybrid assay

The full‐length CDS of *HbCAT2* and *HbSnRK2.6A* were cloned into pGBKT7 and pGADT7 vectors, respectively, resulting in BD‐HbCAT2 and AD‐HbSnRK2.6A plasmids. The yeast transformation and growth were carried out using the Matchmaker system (Clontech Laboratories, Mountain View, CA, USA) according to the manufacturer's protocols. Yeast transformants were selected on double dropout medium lacking Leu and Trp (SD/‐LW), and the protein interaction was determined on quadruple dropout medium lacking Leu, Trp, His and Ade (SD/‐LWHA). Primer sequences are listed in Table S1.

### BiFC assay

The full‐length CDS of *HbSnRK2.6A* and *HbCAT2* were cloned into the pSPYCE or pSPYNE vector containing the C‐terminal half of YFP (cYFP) or the N‐terminal half of YFP (nYFP), producing nYFP‐HbCAT2 and cYFP‐HbSnRK2.6A, respectively. nYFP‐HbCAT2 and cYFP‐HbSnRK2.6A were co‐expressed in *N. benthamiana* leaves for 3 days, and then the YFP (excitation 488 nm, emission 490–550 nm) fluorescence signals were detected by Zeiss LSM980 laser scanning confocal microscope (Zeiss, Oberkochen, Germany) according to our previous report (Lu et al., 2023). Primer sequences are listed in Table S1.

### Co‐IP assays

The full‐length CDS of *HbCAT2* was cloned into the pEGAD vector at the EcoRI site, where the GFP tag was fused to its N‐terminus and under the control of the *35S* promoter. The full‐length CDS of HbSnRK2.6A was cloned into the pCAMBIA1302‐MYC vector, resulting in 35S::HbSnRK2.6A‐MYC. The Co‐IP experiment was performed according to our previously reported methods with some modifications (Lu et al., 2023). Briefly, the empty GFP tag or GFP‐tagged HbCAT2 and MYC‐tagged HbSnRK2.6A were transiently co‐expressed in *N. benthamiana* leaves by *Agrobacterium* injection for 3 days. Total proteins were extracted from the infected leaves by homogenization in IP buffer (150 mM NaCl, 25 mM Tris–HCl, pH 7.5, 0.2% Nonidet P‐40, 1 mM phenylmethylsulfonyl fluoride, and 1 × protease inhibitor cocktail), and then immunoprecipitated by anti‐GFP antibody‐conjugated agarose beads. The resulting precipitants were re‐suspended and detected by anti‐GFP (ABclonal, Wuhan, China; Cat#: AE012) and anti‐MYC (ABclonal, Cat#: AE010) antibodies, respectively.

### GST pull‐down assay

The full‐length CDS of *HbSnRK2.6A* and *HbCAT2* were cloned into the pET28a and pGEX4T‐1 vectors, respectively. The GST pull‐down assay was performed according to our previous reports (Lu et al., 2023). Briefly, GST‐HbCAT2 and 6 × His‐HbSnRK2.6A were expressed and purified in *E. coli*. The GST or GST‐HbCAT2 protein was mixed with the 6 × His‐HbSnRK2.6A protein on ice for 1 h, and then incubated with GST‐Sefinose Resin 4FF (Settled Resin) (Sangon, Shanghai, China; C600031) at 4°C for 3 h. After washing, the eluted protein with Elution Buffer (10 mM GSH in 50 mM Tris–HCl, pH 8.0) was detected with anti‐His (EnoGene Biotech Co., Ltd., New York, NY, USA; #E12‐004‐3) and anti‐GST (ABclonal, AE006) antibodies, respectively. Primer sequences are listed in Table S1.

### Determination of catalase activity

Seedlings were treated with 22°C or 4°C for the indicated time, then sampled and ground to fine powder under liquid nitrogen, and suspended in cold protein extraction buffer (50 mM Tris, 10% glycerol, pH 7.4). After centrifugation at 12 000 ***g*** for 10 min at 4°C, the supernatant was transferred to a new tube for further use. The protein concentration was assayed using the Bradford method, and the catalase (CAT) activity was determined according to our previously described methods (Wang et al., 2022; Yuan, Liu, & Lu, 2017).

### *In vitro* phosphorylation assay

An *in vitro* kinase assay was performed using ATP‐gamma‐S (Abcam, Cambridge, UK; ab92570) according to previous reports (Allen et al., 2007; Zhang et al., 2025). GST‐HbCAT2 and 6 × His‐HbSnRK2.6A proteins incubated in 25 μl kinase buffer (50 mM HEPES (pH 7.5), 5 mM MgCl_2_, 1 mM dithiothreitol (DTT) and 1 mM ATP‐gamma‐S) at room temperature for 30 min. Then, the samples were reacted with 2.5 mM *p*‐Nitrobenzyl mesylate (Abcam, ab138910) at room temperature for 2 h. The phosphorylated proteins were detected by immunoblotting using anti‐thiophosphate ester antibody (Abcam, ab92570). Total protein inputs were visualized by Coomassie brilliant blue (CBB) staining.

### Generation of transgenic plants

To generate *35S::GFP‐HbCAT2* (*HbCAT2‐OE*) transgenic plants, the full‐length CDS of *HbCAT2* was amplified by PCR and cloned into the pEGAD vector at the *EcoR*I site, resulting in pEGAD‐GFP‐*HbCAT2* plasmid. The resulting plasmid was used to transform the Arabidopsis wild‐type plants by *Agrobacterium tumefaciens*‐mediated transformation using the floral‐dip method. Transformants were selected on medium containing Basta, and T4 homozygous transgenic plants were used for our study. To obtain the *HbCAT2‐OE cat2‐1* and *HbCAT2‐OE cbf123* plants, the *HbCAT2‐OE* plants were crossed with the *cat2‐1* and *cbf123* mutants, respectively, and the mutant background was verified by genomic PCR or sequencing.

To create *HbCAT2::GUS* transgenic Arabidopsis plants, the 1865‐bp genomic DNA sequence upstream of the *HbCAT2* translation start site (ATG) was cloned into the pCAMBIA1381‐GUS vector, resulting in an *HbCAT2::GUS* plasmid construct. The resulting plasmid was used to transform the Arabidopsis wild‐type plants by *A. tumefaciens*‐mediated transformation using the floral‐dip method. Transformants were selected on medium containing hygromycin, and T4 homozygous transgenic plants were used for our study. To obtain the *HbCAT2::GUS* *cbf123* and *HbCAT2::GUS* *HbCBF1‐OE* plants, the *HbCAT2::GUS* plants were crossed with the *cbf123* mutant and *HbCBF1‐OE* plants, respectively, and the mutant background was verified by genomic PCR or sequencing.

### 3,3‐Diaminobenzidine and nitroblue tetrazolium staining

The untreated and cold‐treated Arabidopsis seedlings and rubber tree seedling leaves were incubated in freshly prepared DAB staining solution (1 mg ml^−1^ DAB and 0.1% Tween 20 in 10 mM Na_2_HPO_4_) or NBT staining solution (0.1 mg ml^−1^ NBT and 25 mM HEPES buffer, pH 7.6), respectively (Song et al., 2021; Wang et al., 2022), and then rinsed with 70% ethanol several times to remove the chlorophyll. For superoxide anions, the seedlings were vacuum‐infiltrated with 0.1 mg ml^−1^ NBT in 25 mM HEPES buffer (pH 7.6) for 2 h in darkness. Chlorophyll was again removed using 70% ethanol.

### Generation of transgenic *H. brasiliensis* callus

Transgenic *H. brasiliensis* calli were generated as described previously (Luo et al., 2025). Briefly, the CDS of *HbCAT2* was cloned into the binary vector pCAMBIA2300 carrying *GFP* and *NptII* selection markers driven by the 35S promoter (Leclercq et al., 2010). The resulting recombinant plasmid was introduced into *A. tumefaciens* strain EHA105. For inoculation, the transformed EHA105 cells were cultured in liquid LB medium supplemented with 50 mg L^−1^ kanamycin and 25 mg L^−1^ rifampicin until reaching an OD_600_ of 0.6, diluted to an OD_600_ of 0.1–0.2, and used to inoculate precultured calli by brief immersion (5–10 sec). After removing excess bacteria, infected calli were co‐cultured in the dark at 20°C for 3–5 days before being transferred to decontamination medium (SM containing 450 mg L^−1^ timentin). After 42 days, newly developed aggregates were subcultured onto selection medium (DM containing 200 mg L^−1^ paromomycin) (Leclercq et al., 2010). Paromomycin‐resistant calli exhibiting GFP fluorescence were selected as stable transgenic lines. Quantitative real‐time PCR (qRT‐PCR) was performed to further identify the transgenic *H. brasiliensis* callus lines.

### Statistical analysis

All experiments were performed with at least three independent biological replicates. Biological replicates were defined as independently prepared experimental units, including plant materials and callus cultures. Data are presented as mean ± standard deviation (SD), as indicated in figure legends. Statistical analyses were performed using GraphPad Prism (version 8.0). Differences between groups were assessed using Student's *t*‐test for pairwise comparisons or one‐way anova followed by Tukey's *post hoc* test for multiple comparisons, with significance set at *P* < 0.05.

## AUTHOR CONTRIBUTIONS

H‐MY and W‐CL conceived and designed the research. R‐FS performed the experiments with the help of WW, LL, and HY. R‐FS analyzed the data and prepared the figures. CT interpreted the results and revised the article. W‐CL and H‐MY wrote the article.

## CONFLICT OF INTEREST

All authors declare that they have no competing interests.

## Supporting information


**Figure S1.** Phylogenetic analysis of HbCAT2 and other plant catalase proteins.
**Figure S2.** The transcription, protein and phosphorylation levels of HbCAT2 in *H. brasiliensis* treated with cold.
**Figure S3.** The transcription and protein levels of HbCAT2 in transgenic *H. brasiliensis HbCAT2‐OE* calli.
**Figure S4.** *HbCAT2* overexpression improves the cold tolerance in Arabidopsis.
**Figure S5.** HbCBF1 activates AtCAT2 promoter activity.
**Figure S6.** Disruption of CAT2 impairs HbCBF1‐mediated cold tolerance.
**Figure S7.** Overexpression of *HbCAT2* improves cold tolerance of the Arabidopsis *cbf123* mutant.
**Figure S8.** HbSnRK2.6A interacts with AtCAT2.
**Figure S9.** HbSnRK2.6A phosphorylates HbCAT2 at its Ser‐347 and Ser‐437 sites.

**Table S1.** List of the primers used in this study.

**Table S2.** Gene sequences used in this study.

**Table S3.** Uncropped blot and gel images in this study.

## Data Availability

Data that support the findings of this study are available within the article and its Supporting Information files. Gene sequences used in this study are listed in the Table S2. Uncropped blot and gel images in this study were provided in Table S3.
