# Supplementary material for: Transcription and phosphorylation of CATALASE2 contribute to cold stress tolerance in Hevea brasiliensis
Source: Plant J. 2026 Aug 28;127(4):e71097. doi: 10.1111/tpj.71097 (PMC13522878; doi:10.1111/tpj.71097)

**Supplemental Table S3.** Uncropped blot and gel images in this study.

| Figure No. | Uncropped images |
|---|---|
| Figure 3C | Figure 3C 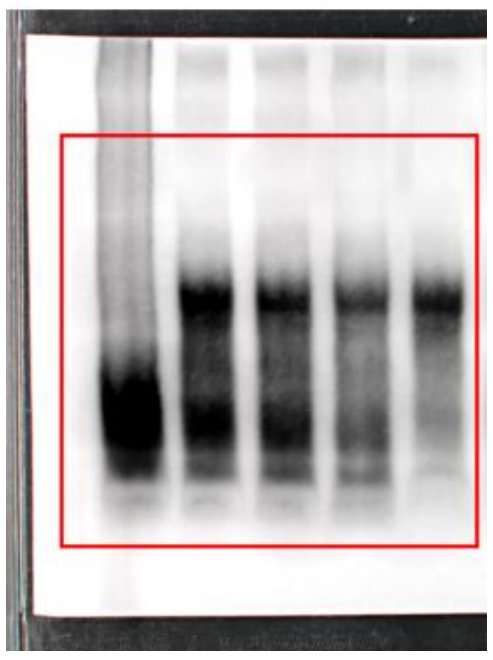 |
| Figure 4C | Figure 4C<br>Input (GFP) IP (GFP) Input (MYC) IP (MYC) 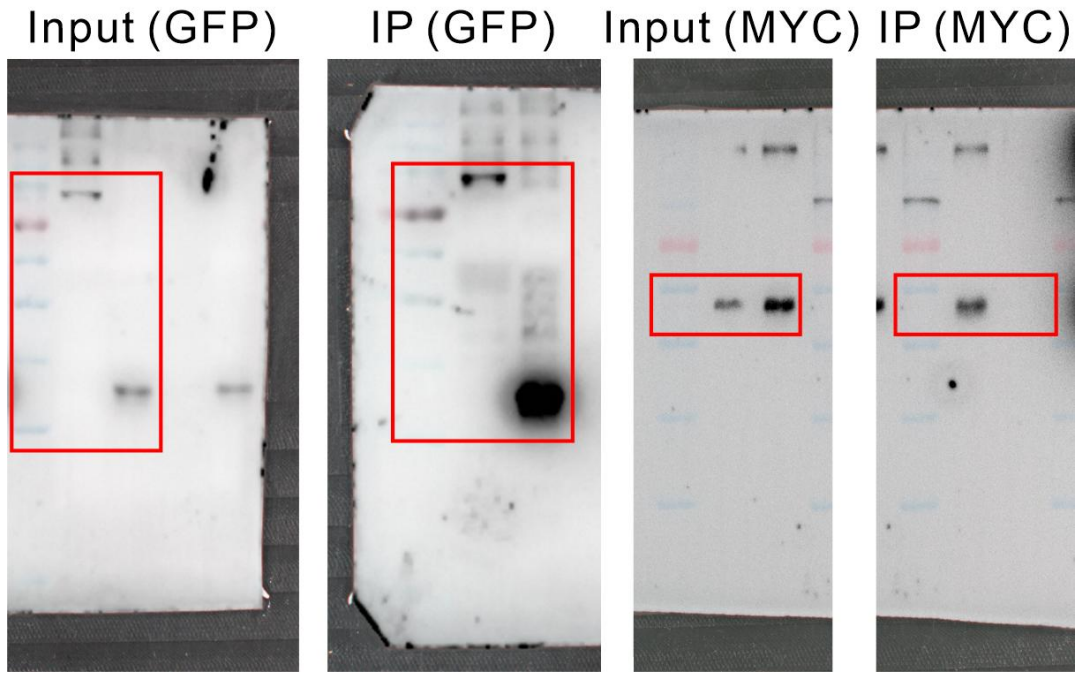 |
| Figure 4D | Figure 4D<br>Input (GST) PD (GFP) Input (His) PD (MYC) 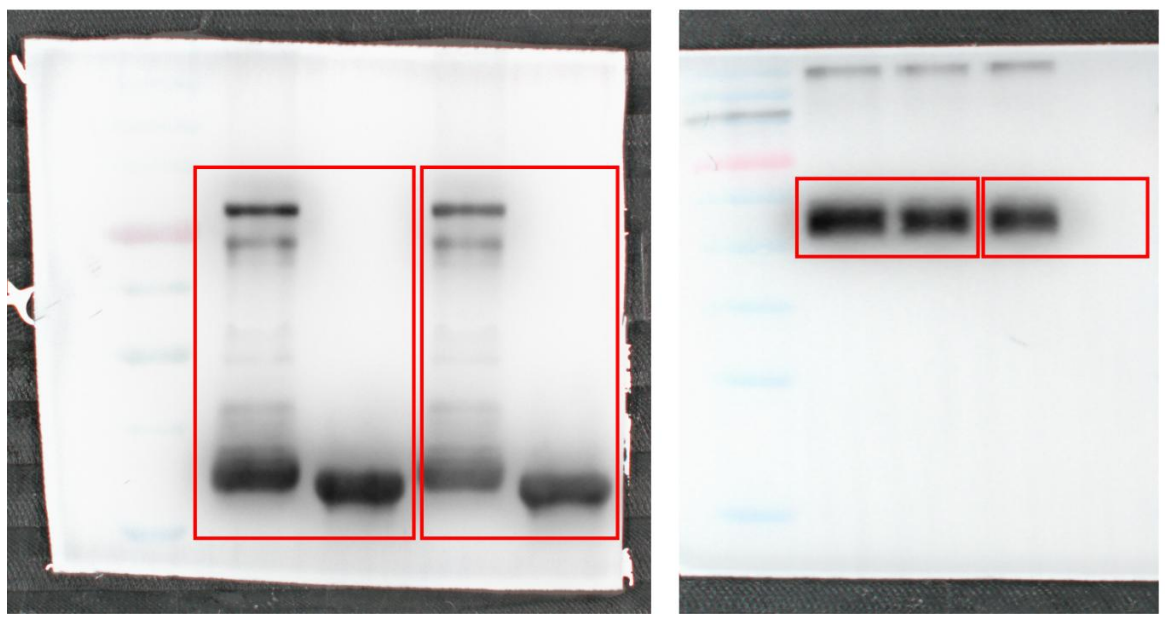 |
| Figure 4E | Figure 4E<br>anti-thiophos-phaste ester CBB 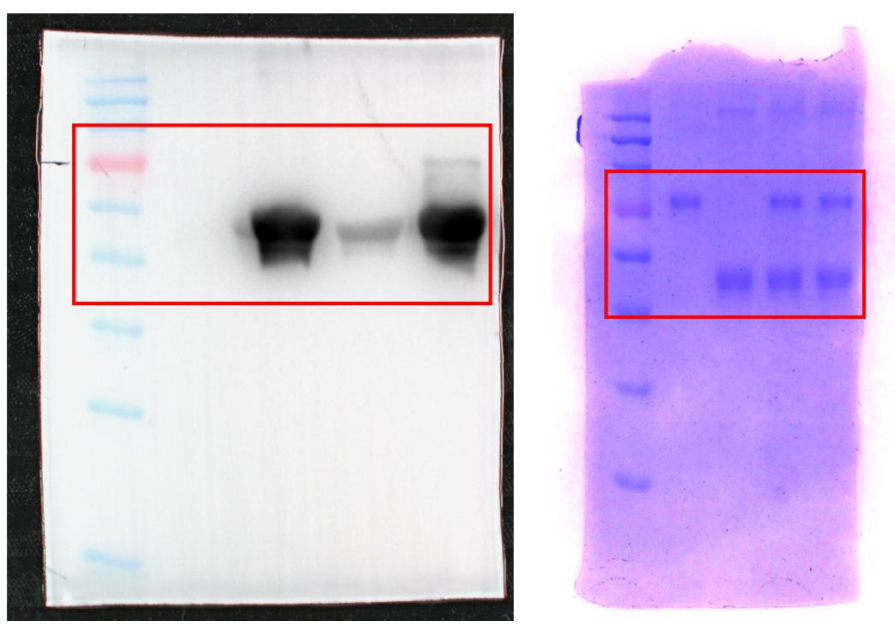 |

Figure 4F

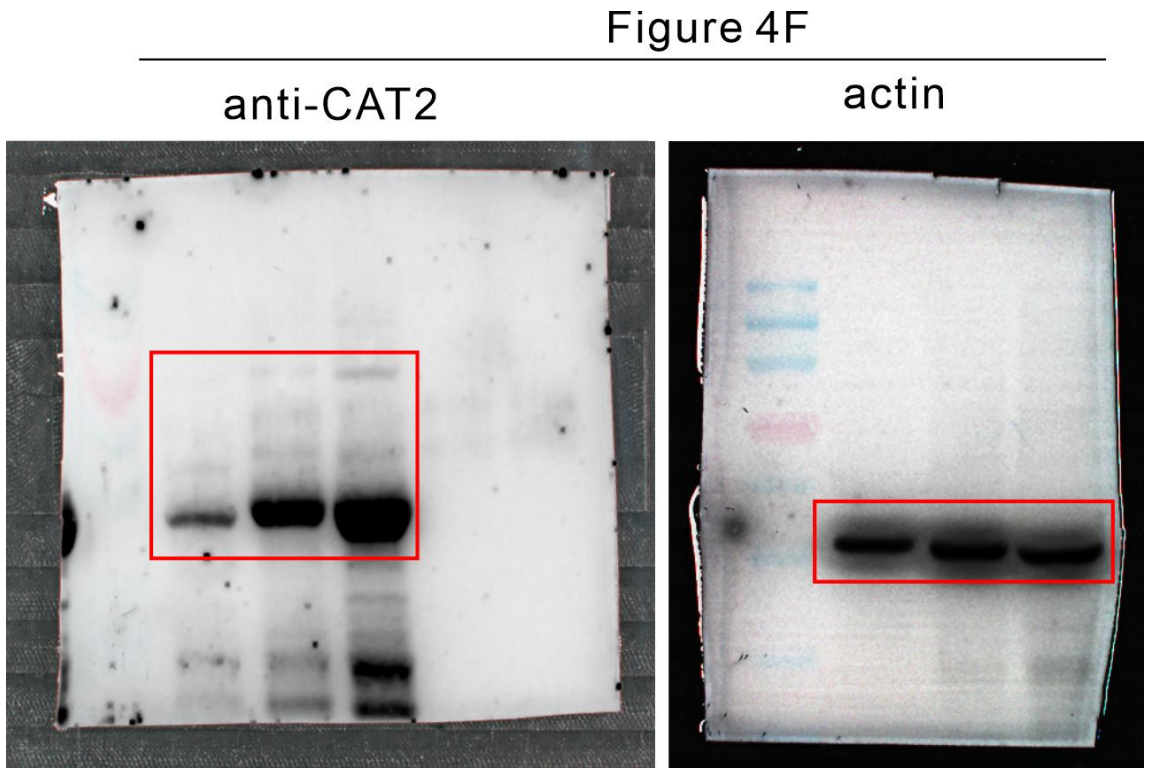


Figure S2

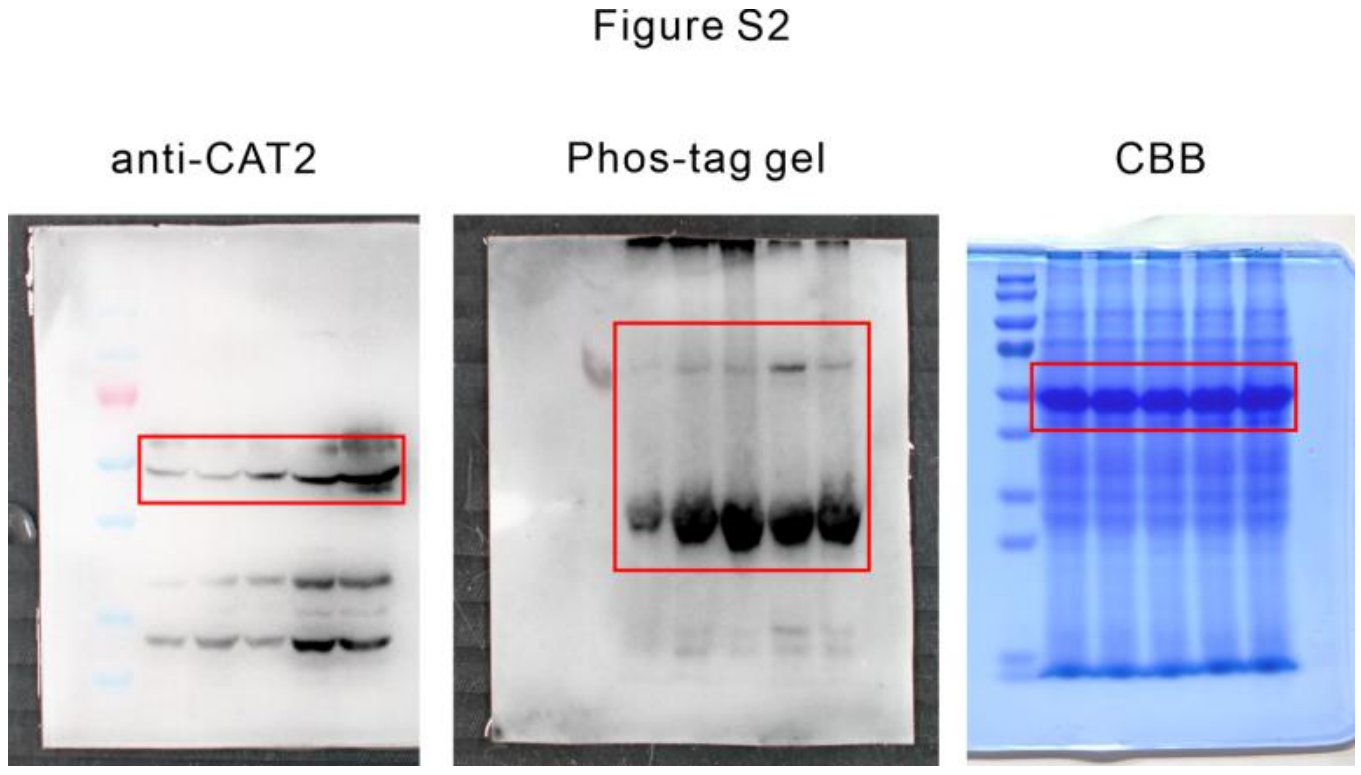


Figure S3

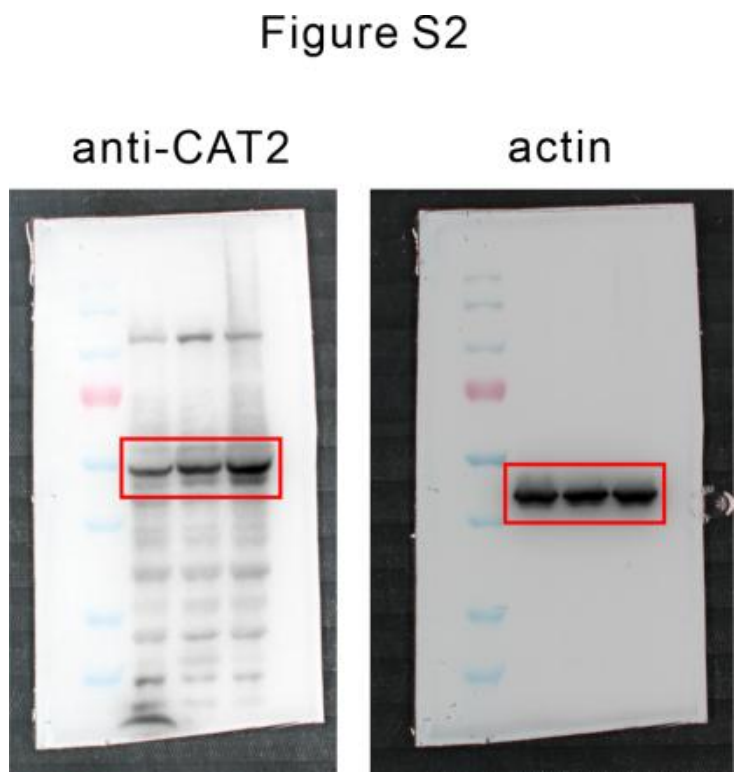


Figure S8

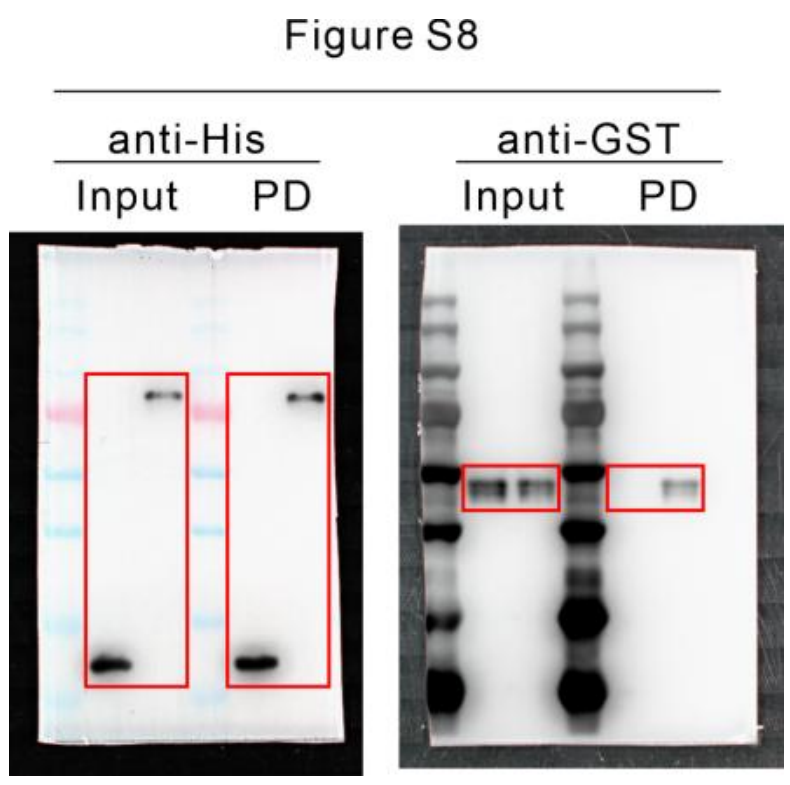

Figure S9

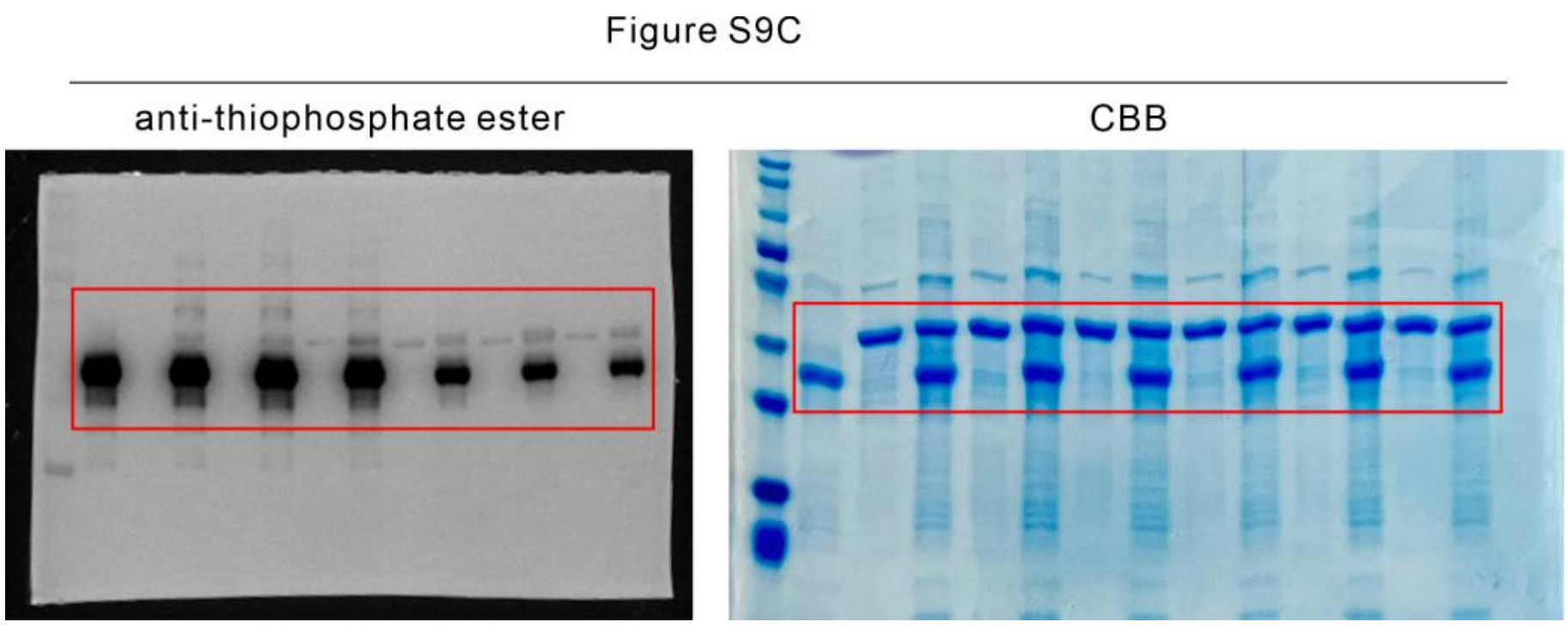

Supplement: Supplementary file 1 [file TPJ-127-0-s001.pdf]
