# Supplementary material for: Transcription and phosphorylation of CATALASE2 contribute to cold stress tolerance in Hevea brasiliensis
Source: Plant J. 2026 Aug 28;127(4):e71097. doi: 10.1111/tpj.71097 (PMC13522878; doi:10.1111/tpj.71097)
Supplement: Supplementary file 2 [file TPJ-127-0-s002.pdf]

Supplemental Table S1. List of the primers used in this study.

Primer name	Sequence (5’ to 3’)
**Primers used for molecular cloning**	
1300-35S-HbCAT2-F	CGAGCTGTACAAGTACCTGCAGATGGATCCCTACAAGAACCG
1300-35S-HbCAT2-R	gatacgaacgaaagctCTGCAGTCAAATGCTTGGCCTCGC
1300-YCE-HbCAT2-F	CGAGCTGTACAAGTCACTGCAGATGGATCCCTACAAGAACCG
1300-YCE-HbCAT2-R	gatgatacgaacgaaagctCTGTCAAATGCTTGGCCTCGC
0800-HbCAT2pro-F	TCCTGCAGCCCGGGGGAGAGATTGAGTGTTCGAGCAG
0800-HbCAT2pro-R	CGCTCTAGAACTAGTGGAGCAAGAGATATTTGTGAATTTTTGATGGG
1381Z-HbCAT2pro-F	GATTACGAATTCCCGGGGATCGAGATTGAGTGTTCGAGCAG
1381Z-HbCAT2pro-R	CTGCAGGTCGACGGATCCAGCAAGAGATATTTGTGAATTTTTGATGGG
pAbAi-CAT2pro-F	CCGGGGATCTGTCGACCGTGCTGTACAATTTCACTTGTG
pAbAi-CAT2pro-R	CATGCCTCGAGGTCGACCGTCGCTTGCACGTCC
pGBK-HbCAT2-N-F	GGCCGAATTCCCGGGGATGGATCCCTACAAGAACCG
pGBK-HbCAT2-N-R	CGCTGCAGGTCGACGATTGGTTCTTCCAACCTTGACTG
pGBK-HbCAT2-C-F	GGCCGAATTCCCGGGGCATAGCCATGCCACCCAG
pGBK-HbCAT2-C-R	CGCTGCAGGTCGACGAATGCTTGGCCTCGCATTG
pET28a-HbCAT2-F	AGCAAATGGGTCGCGGAATGGATCCCTACAAGAACCG
pET28a-HbCAT2-R	CGGAGCTCGAATTCGGATCAAATGCTTGGCCTCGC
HbCAT2pro-F	GAGATTGAGTGTTCGAGCAG
HbCAT2pro-R	AGCAAGAGATATTTGTGAATTTTTGATGGG
HbCAT2pro-600bp-F	GAAGCATTATTGTAATCTTCAATTTAATG
HbCAT2pro-877bp-F	CATCCAGTCCTGCATAAATATCGTCGTGC
HbCAT2pro-1028bp-F	GGAGCCATTACCTGGTGACGTTTTCAATTG
HbCAT2-atg-R	GCGCTTGATGGACGGTTCTTGTAGGGATCCAT
pET28a-HbSnRK2.6-F	AGCAAATGGGTCGCGGAATGGATCGTTCGGCGG
pET28a-HbSnRK2.6-R	CGGAGCTCGAATTCGGATCACATTGCATATACTATCTCTCCAC
PET28a-HbCBF1-F	AGCAAATGGGTCGCGGAATGGATGTTTTCCCTCAATATTCTG
PET28a-HbCBF1-R	CGGAGCTCGAATTCGGATTATATAGAAAAACTCCATAGTGACACATCAGC
pGAD-HbCBF1-F	GGGCATCGATACGGGATGGATGTTTTCCCTCAATATTCTGACTC
pGAD-HbCBF1-R	AGCTCGAGCTCGATGTTATATAGAAAAACTCCATAGTGACACATCAGC
pEGAD-HbCBF1-F	CGAGAAGCTTGGATCCATGGATGTTTTCCCTCAATATTCTGACTC
pEGAD-HbCBF1-R	TATCTAGATCCGGTGGATTATATAGAAAAACTCCATAGTGACACATCAGC
pGEX4T-HbCBF1-F	ATCCCCGGAATTCCCGATGGATGTTTTCCCTCAATATTCTGACTC
pGEX4T-HbCBF1-R	CGCTCGAGTCGACCCGGTTATATAGAAAAACTCCATAGTGACACATCAGC
YNE/YCE-HbCBF1-F	CTCGGTACCCGGGGATCCATGGATGTTTTCCCTCAATATTCTGACTC
YNE/YCE-HbCBF1-R	GTCGACTCTAGAGGATCCTATAGAAAAACTCCATAGTGACACATCAGC
**Probes for EMSA**	
HbCAT2-BioF	Bio-GGCGAGATGTGAAATTACCGACAGTAAAATTTTAAATTTATC
HbCAT2-F	GGCGAGATGTGAAATTACCGACAGTAAAATTTTAAATTTATC

HbCAT2-R	GATAAATTTAAAATTTTACTGTCGGTAATTTCACATCTCGCC
mutHbCAT2-F	GGCGAGATGTGAAATTACAAACAGTAAAATTTTAAATTTATC
mutHbCAT2-R	GATAAATTTAAAATTTTACTGTTTGTAATTTCACATCTCGCC
**Primers used for RT-qPCR**	
Hb18S-F	CCATAAACGATGCCGACCAG
Hb18S-R	CAGCCTTGCGACCATACTC
HbCAT2-F	TTCGTCCTTGACTGTTGGGC
HbCAT2-R	CATGGACAACACGCTCTGGA
