# Supplementary material for: Transcription and phosphorylation of CATALASE2 contribute to cold stress tolerance in Hevea brasiliensis
Source: Plant J. 2026 Aug 28;127(4):e71097. doi: 10.1111/tpj.71097 (PMC13522878; doi:10.1111/tpj.71097)
Supplement: Supplementary file 3 [file TPJ-127-0-s003.pdf]

Supplemental Figures

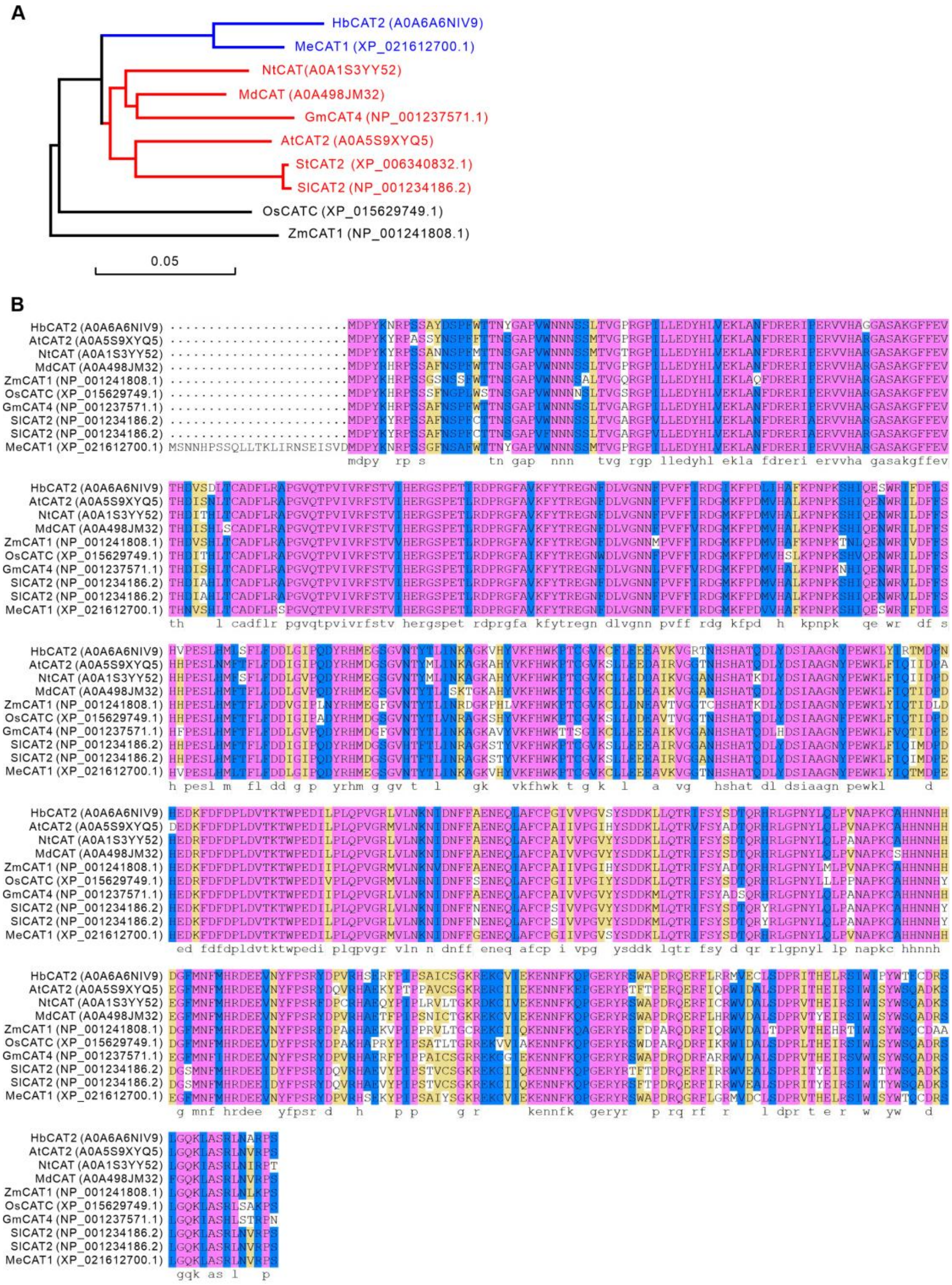

Figure S1. Phylogenetic analysis of HbCAT2 and other plant catalase proteins.

(**A**) Phylogenetic analysis of HbCAT2 from *H. brasiliensis* and other plant species using the neighbor-joining method. GenBank accessions of the predicted HbCAT2 amino acid sequences used are as follows: HbCAT2 (A0A6A6NIV9), AtCAT2 (A0A5S9XYQ5), NtCAT (A0A1S3YY52), MdCAT (A0A498JM32), ZmCAT1 (NP_001241808.1), OsCATC (XP_015629749.1), GmCAT4 (NP_001237571.1), SlCAT2 (NP_001234186.2), and MeCAT1 (XP_021612700.1).

(**B**) Sequence alignment of catalase proteins from *H. brasiliensis* and other plant species shown above.

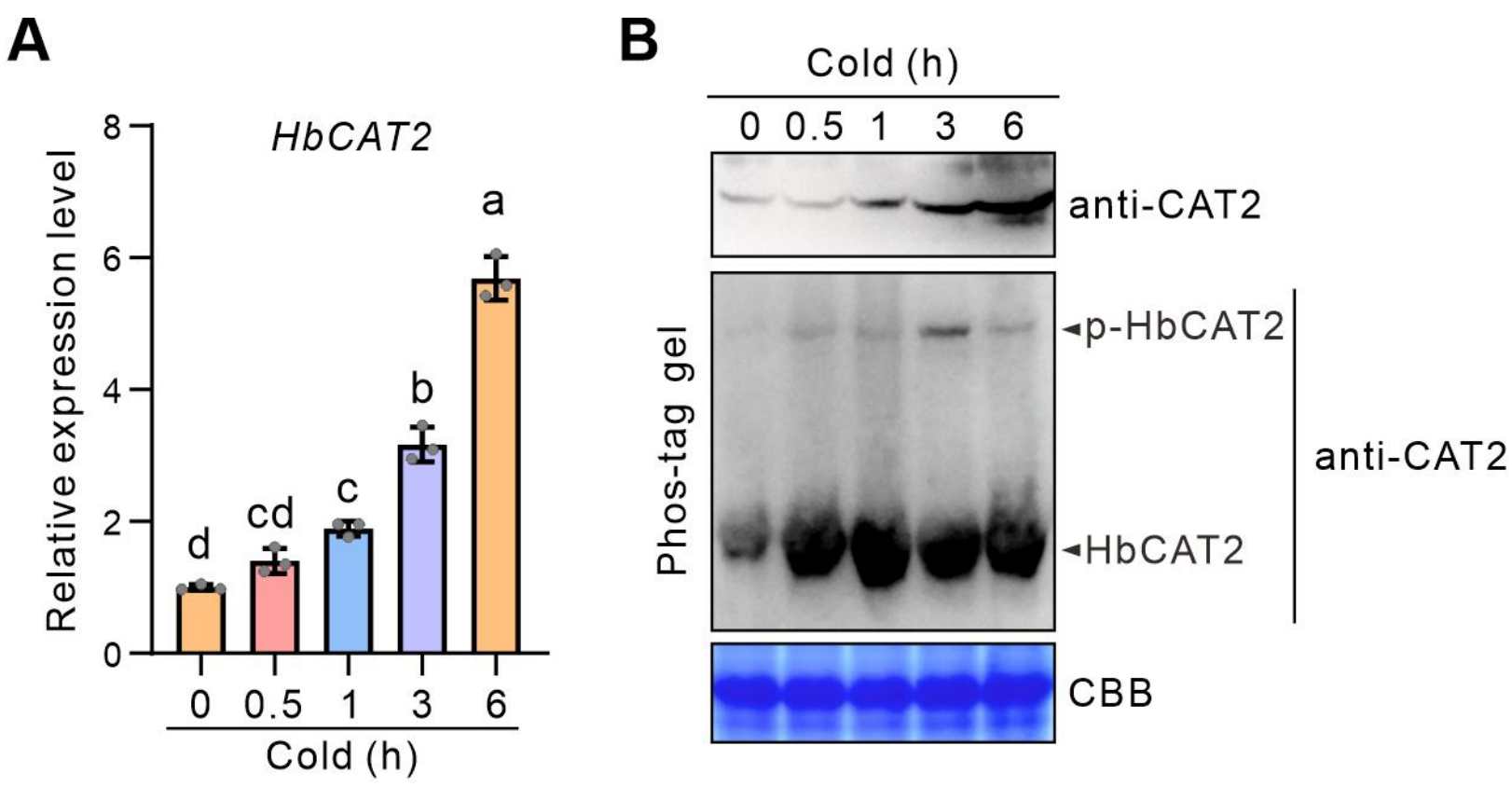

Figure S2. The transcription, protein and phosphorylation levels of HbCAT2 in *H. brasiliensis* treated with cold.

(A) Expression levels of *HbCAT2* in *H. brasiliensis* seedling leaves response to cold temperatures. The *H. brasiliensis* seedlings were treated with 10°C for 0 h, 0.5 h, 1 h, 3 h and 6 h, and then the leaves were collected for total RNA extraction and RT-qPCR analysis. *HbeIF2* was used as an internal control. Data are means ± SD (n = 3). Bars with different letters indicate significant differences at $P < 0.05$, determined using ANOVA with a Tukey's multiple comparison test.

(B) HbCAT2 protein levels and phosphorylation in *H. brasiliensis* seedlings treated with cold. Total proteins extracted from the *H. brasiliensis* seedlings treated with cold (10°C) for 0 h, 0.5 h, 1 h, 3 h and 6 h were separated by SDS-PAGE gel and phos-tag gel, and then detected by anti-CAT2 antibody. Coomassie Brilliant Blue (CBB) staining was used as the loading control.

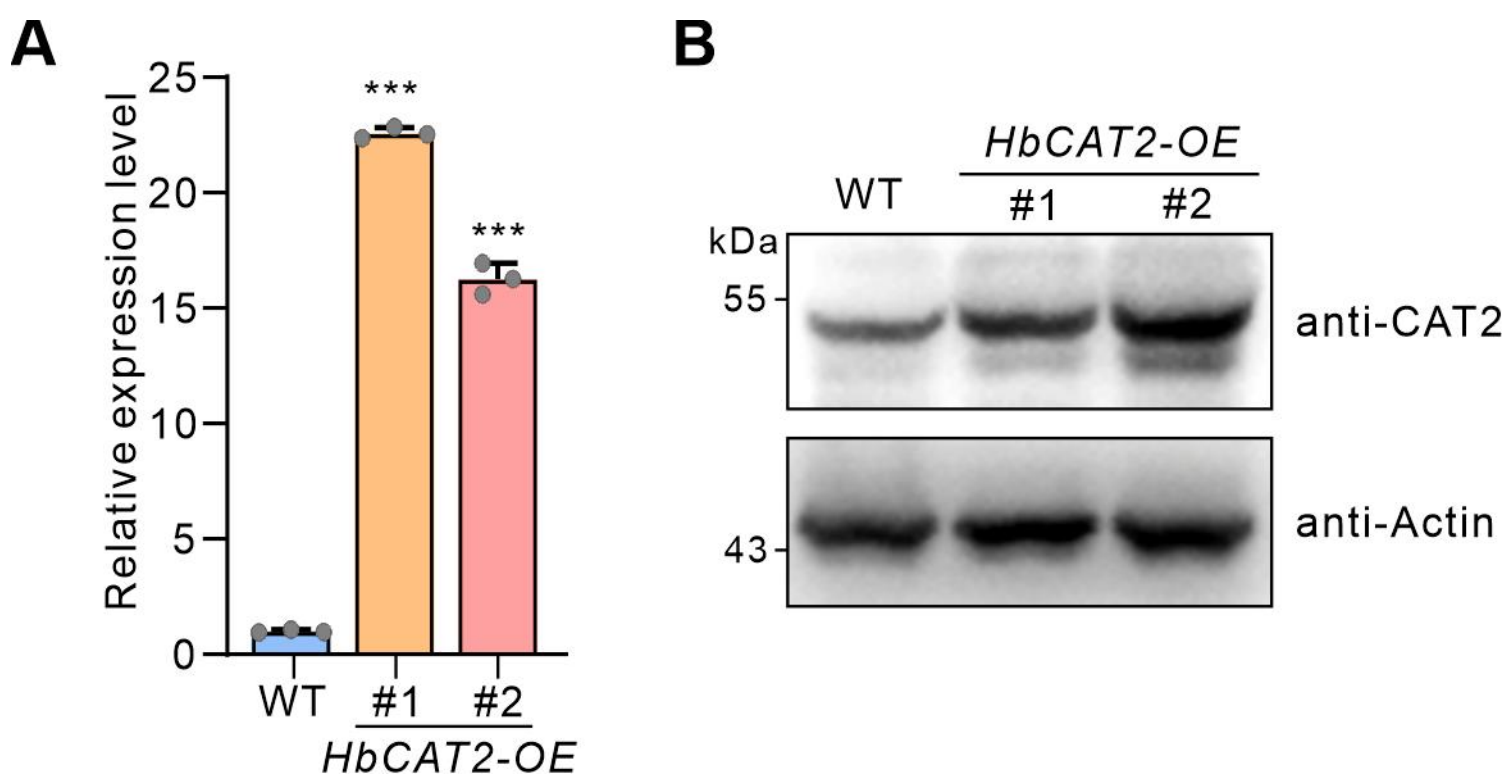

Figure S3. The transcription and protein levels of HbCAT2 in transgenic *H. brasiliensis HbCAT2-OE* calli.

(**A**) Relative expression levels of *HbCAT2* in the wild-type and transgenic *H. brasiliensis HbCAT2-OE* calli. *Hb18S* was used as an internal control. Data are means ± SD (n = 3). Statistical significance is indicated by asterisks (Student's *t* test; ****P* < 0.001).

(B) HbCAT2 protein levels in the wild-type and transgenic *H. brasiliensis HbCAT2-OE* calli. Actin was used as the loading control.

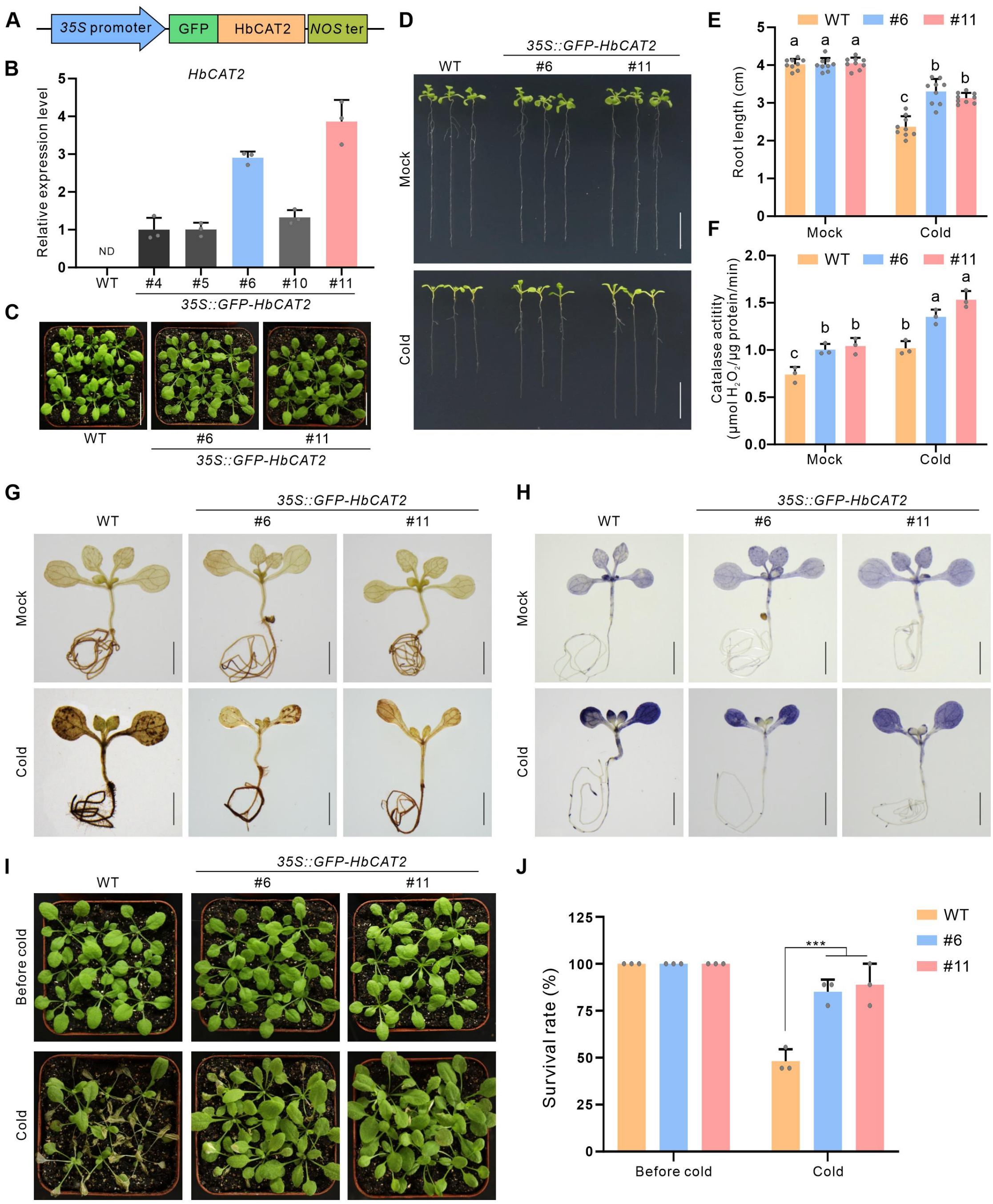

Figure S4. *HbCAT2* overexpression improves the cold tolerance in Arabidopsis.

(**A-C**) A diagram showing the construction of 35S::GFP-HbCAT2 (A), and the expression levels of HbCAT2 in the *35S::GFP-HbCAT2* transgenic Arabidopsis plants were determined using RT-qPCR (B). The phenotype of 3-week-old wild-type and two independent *35S::GFP-HbCAT2* transgenic lines were shown in (C). Bar = 2.5 cm.

(**D-E**) Root growth phenotype (D) of the wild-type and *35S::GFP-HbCAT2* plant. 5-d-old wild-type and *35S::GFP-HbCAT2* plant seedlings were treated with 22°C or 10°C for 7 d, and then the root lengths were measured (E). Data are means ± SD (n = 9 from three biological replicates). Bars with different letters indicate significant differences at $P < 0.05$, determined using ANOVA with a Tukey's multiple comparison test. Bar = 1 cm.

(**F**) Catalase activities were determined in (D). Data are means ± SD (n = 3). Bars with different letters indicate significant differences at $P < 0.05$, determined using ANOVA with a Tukey's multiple comparison test.

(**G-H**) DAB (G) and NBT (H) staining images of the wild-type and *35S::GFP-HbCAT2* plants treated with normal temperature and cold. 5-d-old wild-type and *35S::GFP-HbCAT2* plant seedlings were treated with 22°C or 4°C for 3 d, and then subjected to DAB or NBT staining. Bar = 0.5 cm.

(**I-J**) Phenotype (I) and survival rate (J) of the wild-type and *35S::GFP-HbCAT2* plants treated with freezing stress. 3-week-old wild-type and *35S::GFP-HbCAT2* plants grown in soil were cold acclimated at 4°C for 2d, then put into a freezing chamber at 0°C, with the temperature dropping by 1°C per hour until -8°C. The seedlings were kept at 4°C for 12 h in the dark and grown for an additional 3 d at 22°C. Data are means ± SD (n = 3). Statistical significance is indicated by asterisks (Student's *t* test; ***$P < 0.001$).

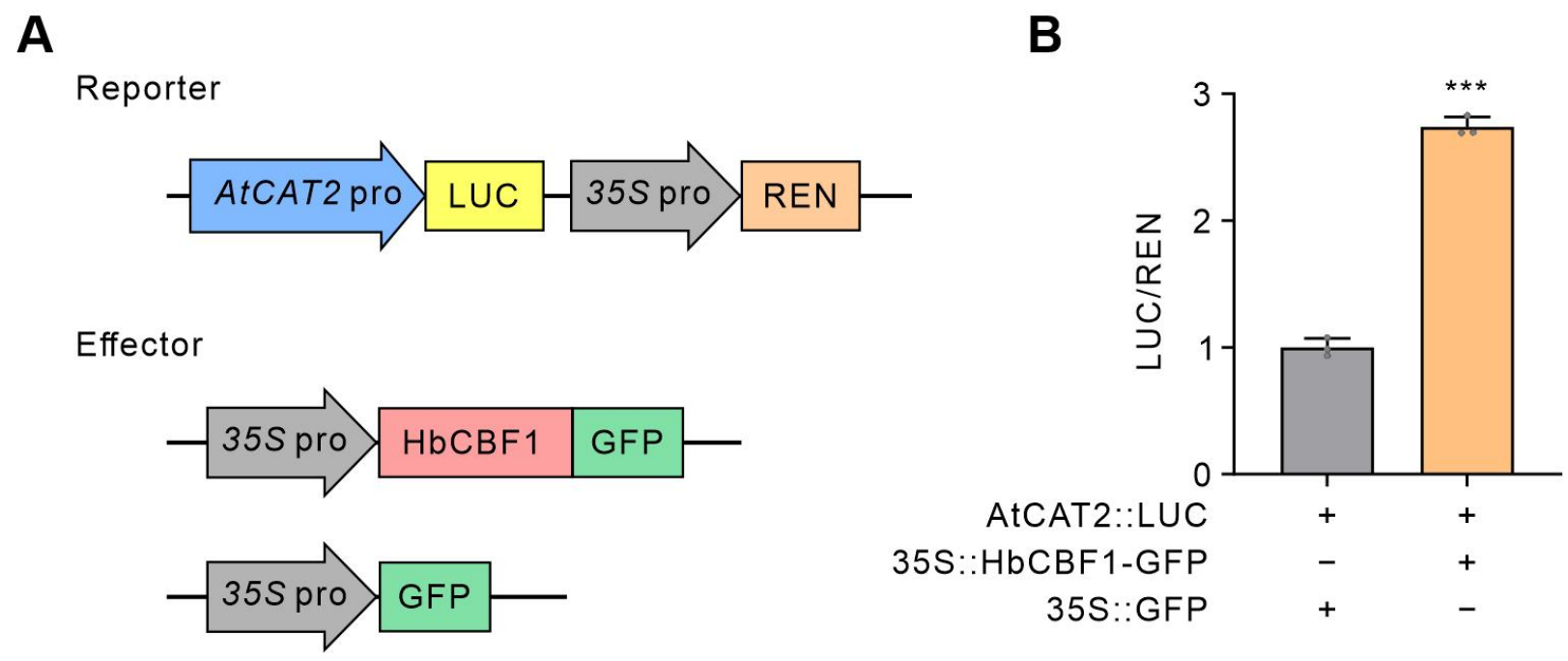

Figure S5. HbCBF1 activates AtCAT2 promoter activity.

(**A-B**) A diagram (A) showing the reporter (AtCAT2::LUC) and the effectors (35S::HbCBF1-GFP and GFP), respectively. AtCAT2::LUC was co-expressed with 35S::HbCBF1-GFP or GFP in the *N. benthamiana* leaves, and then the activities of both LUC and REN were determined. The ratio of LUC to REN was calculated (B). Data are means ± SD (n = 3). Statistical significance is indicated by asterisks (Student's *t* test; ****P* < 0.001).

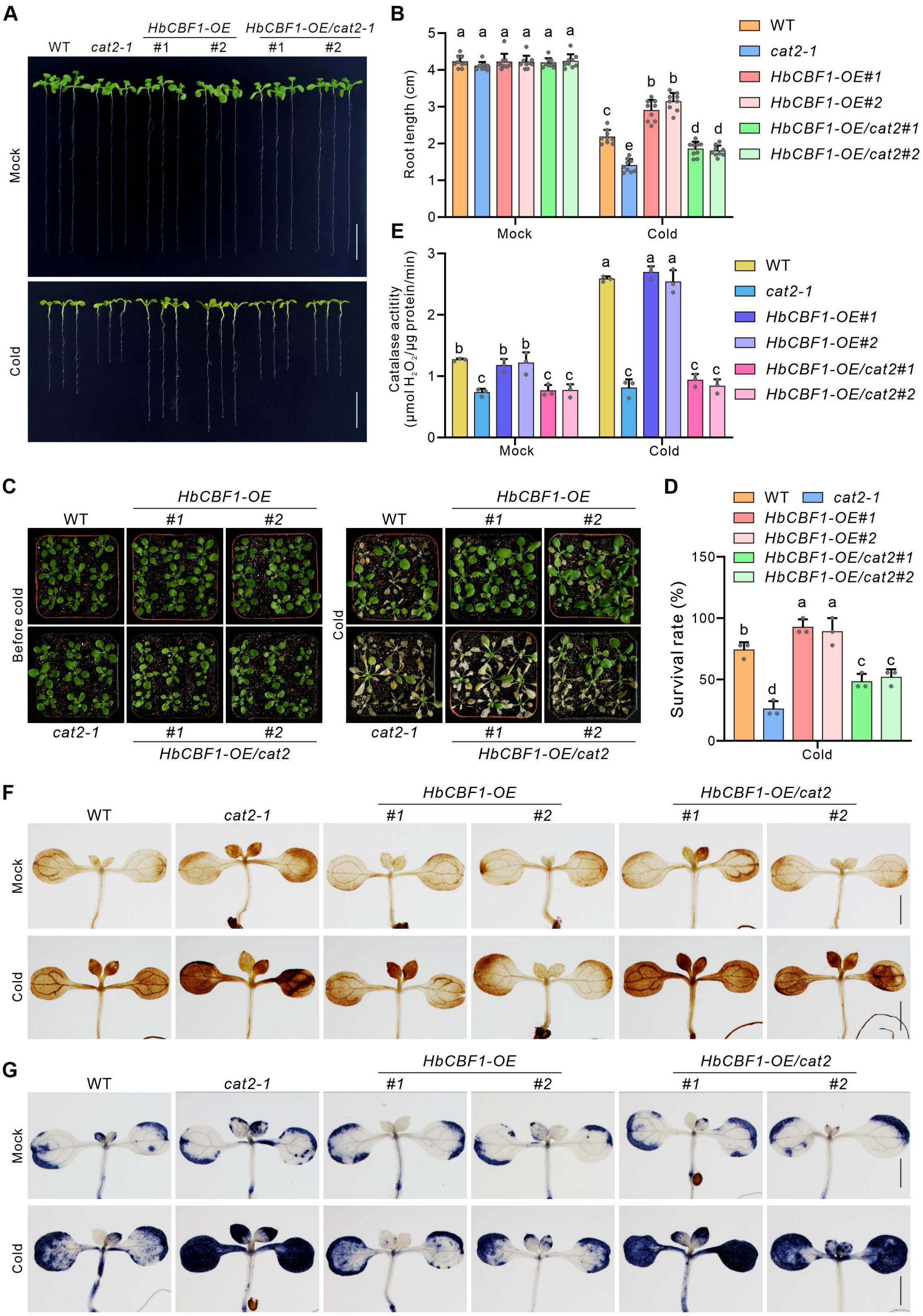

Figure S6. Disruption of CAT2 impairs HbCBF1-mediated cold tolerance.

(**A-B**) Root growth phenotype (A) of the wild-type, *cat2-1*, *35S::HbCBF1-GFP* (*HbCBF1-OE*) and *35S::HbCBF1-GFP/cat2-1* (*HbCBF1-OE/cat2-1*) plants. 5-d-old wild-type, *cat2-1*, *HbCBF1-OE* and *HbCBF1-OE/cat2-1* seedlings were treated with 22°C or 10°C for 7 d, and then the root lengths were measured (B). Data are means ± SD (n = 9 from three biological replicates). Bars with different letters indicate

significant differences at *P* < 0.05, determined using ANOVA with a Tukey's multiple comparison test. Bar = 1 cm.

(**C-D**) Phenotype (C) and survival rate (D) of the wild-type, *cat2-1*, *HbCBF1-OE* and *HbCBF1-OE/cat2-1* plants treated with freezing stress. 3-week-old wild-type, *cat2-1*, *HbCBF1-OE* and *HbCBF1-OE/cat2-1* plants grown in soil were cold acclimated at 4°C for 2d, then put into a freezing chamber at 0°C, with the temperature dropping by 1°C per hour until -8°C. The seedlings were kept at 4°C for 12 h in the dark and grown for an additional 3 d at 22°C. Data are means ± SD (n = 3). Bars with different letters indicate significant differences at *P* < 0.05, determined using ANOVA with Tukey's multiple comparison test.

(**E**) Catalase activities were determined in (A). Data are means ± SD (n = 3). Bars with different letters indicate significant differences at *P* < 0.05, determined using ANOVA with Tukey's multiple comparison test.

(**F-G**) DAB (F) and NBT (G) staining images of the wild-type, *cat2-1*, *HbCBF1-OE* and *HbCBF1-OE/cat2-1* plants treated with normal temperature and cold. 5-d-old wild-type, *cat2-1*, *HbCBF1-OE* and *HbCBF1-OE/cat2-1* plant seedlings were treated with 22°C or 4°C for 3 d, and then subjected to DAB or NBT staining. Bars = 0.5 cm.

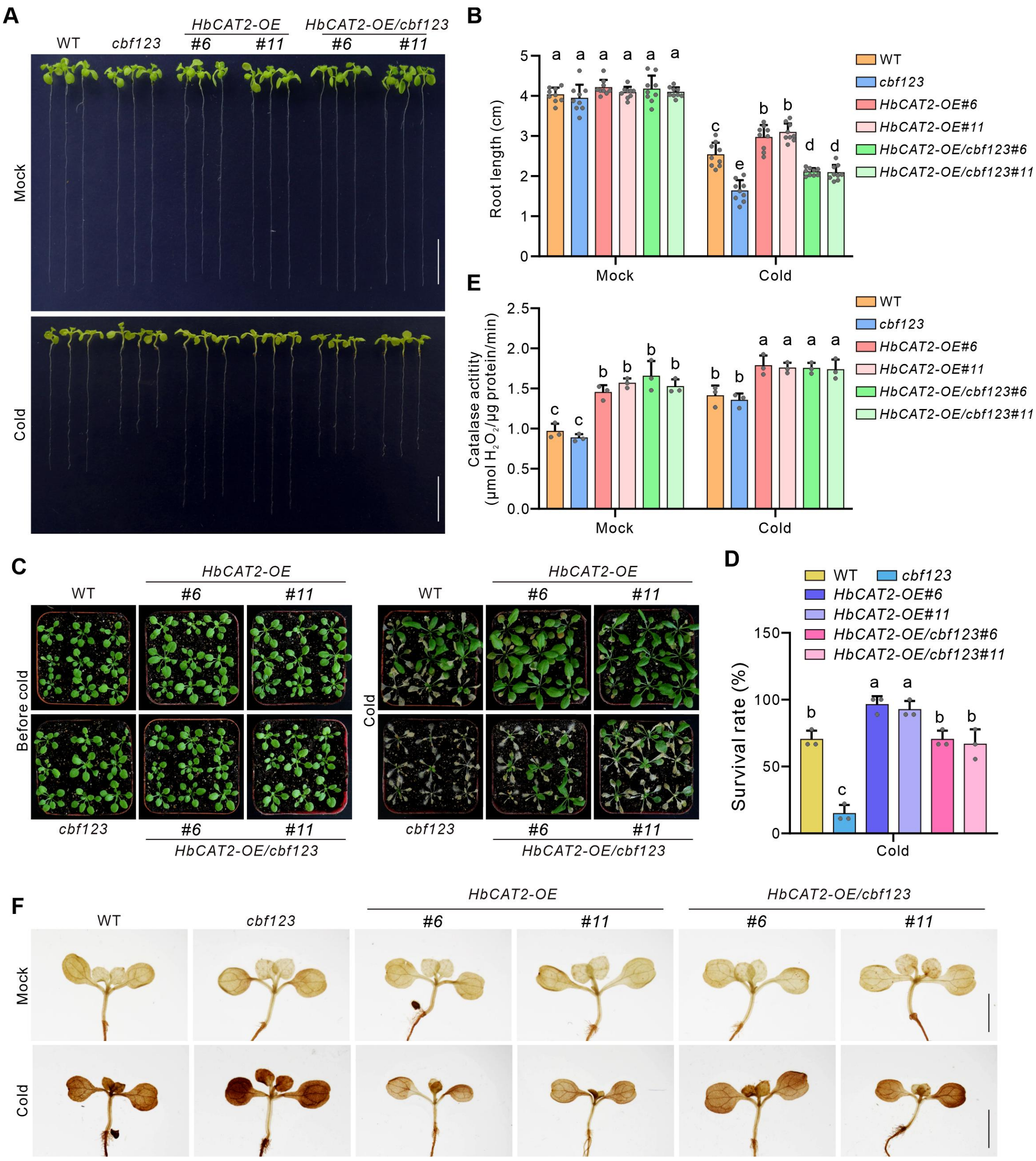

Figure S7. Overexpression of *HbCAT2* improves cold tolerance of the Arabidopsis *cbf123* mutant.

(**A-B**) Root growth phenotype (A) of the wild-type, *cbf123*, *35S::HbCAT2-GFP* (*HbCAT2-OE*) and *35S::HbCAT2-GFP/cbf123* (*HbCAT2-OE/cbf123*) plants. 5-d-old wild-type, *cbf123*, *HbCAT2-OE* and *HbCAT2-OE/cbf123* plant seedlings were treated with 22°C or 10°C for 7 d, and then the root lengths were measured (B). Data are means ± SD (n = 9 from three biological replicates). Bars with different letters indicate significant differences at $P < 0.05$, determined using ANOVA with a Tukey's multiple comparison test. Bar = 1 cm.

(**C-D**) Phenotype (C) and survival rate (D) of the wild-type, *cbf123*, *HbCAT2-OE* and *HbCAT2-OE/cbf123* plants treated with freezing stress. 3-week-old wild-type, *cbf123*, *HbCAT2-OE* and *HbCAT2-OE/cbf123* plants grown in soil were cold acclimated at 4°C for 2d, then transferred into a freezing chamber at 0°C, with the temperature dropping by 1°C per hour until -8°C. The seedlings were kept at 4°C for 12 h in the

dark and grown for an additional 3 d at 22°C. Data are means ± SD (n = 3). Bars with different letters indicate significant differences at $P < 0.05$, determined using ANOVA with Tukey's multiple comparison test.

(**E**) Catalase activities were determined in (A). Data are means ± SD (n = 3). Bars with different letters indicate significant differences at $P < 0.05$, determined using ANOVA with Tukey's multiple comparison test.

(**F**) DAB staining images of the wild-type, *cbf123*, *HbCAT2-OE* and *HbCAT2-OE/cbf123* plants treated with normal temperature and cold. 5-d-old wild-type, *cbf123*, *HbCAT2-OE* and *HbCAT2-OE/cbf123* seedlings were treated with 22°C or 4°C for 3 d, and then subjected to DAB staining. Bars = 0.5 cm.

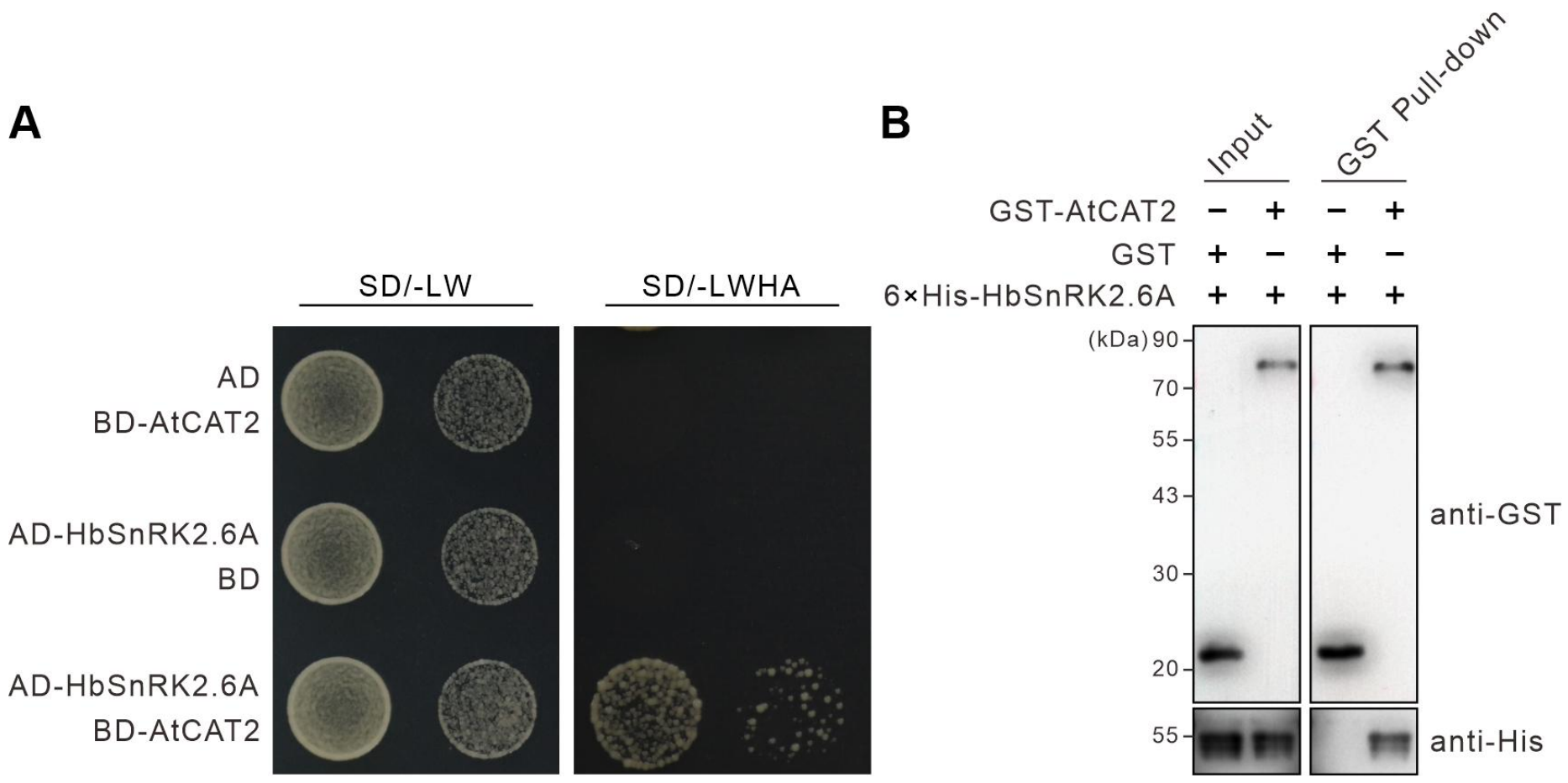

Figure S8. HbSnRK2.6A interacts with AtCAT2.

(**A**) Yeast two-hybrid assay showing the interaction of HbSnRK2.6A with AtCAT2.

(B) The *in vitro* interaction between AtCAT2 and HbSnRK2.6A was determined by GST pull-down. The purified GST or GST-tagged AtCAT2 was incubated with 6×His-tagged HbSnRK2.6A, and the mixture was pulled down by GST beads. The eluted proteins were detected with anti-GST and anti-His antibodies, respectively.

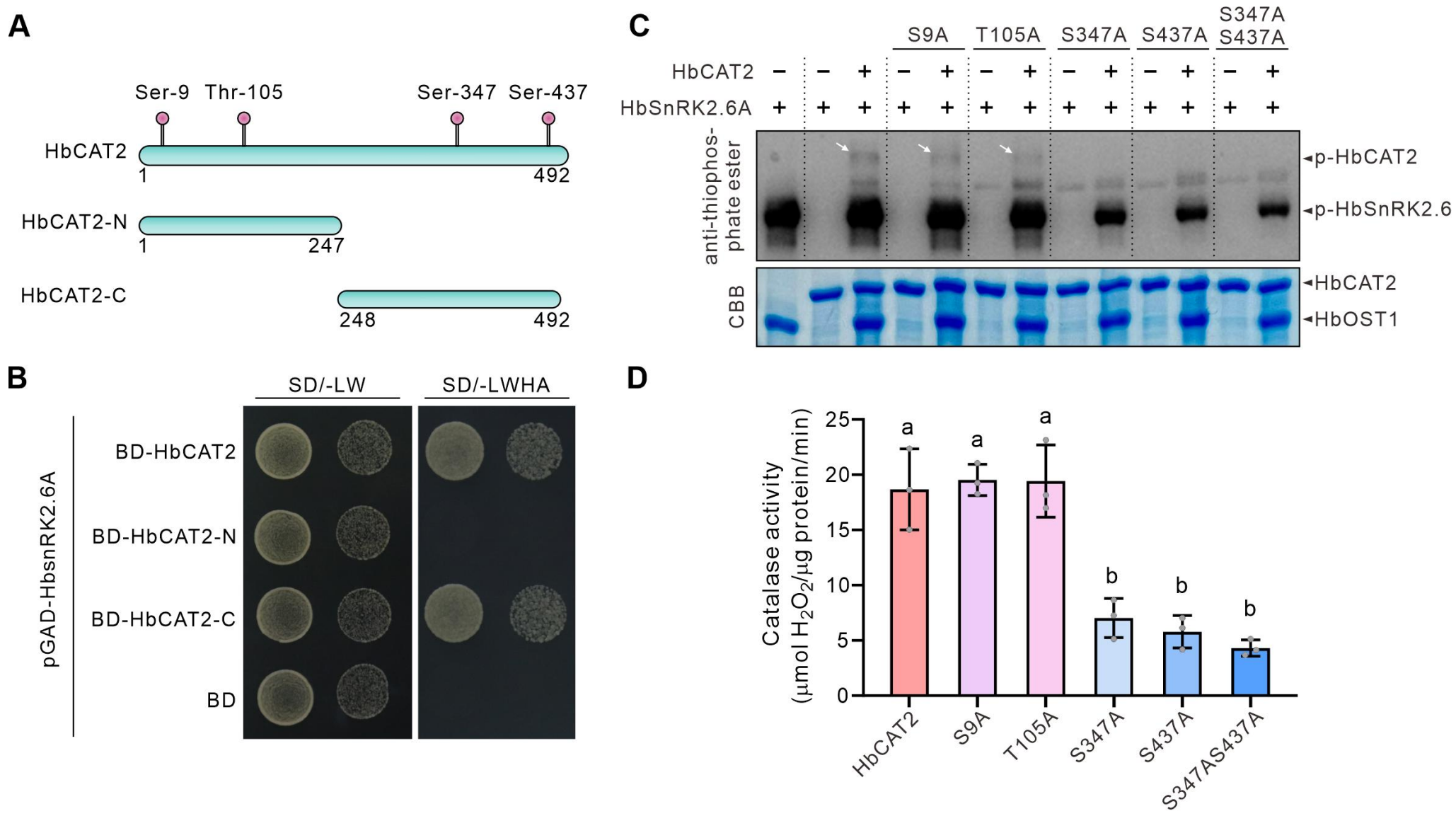

Figure S9. HbSnRK2.6A phosphorylates HbCAT2 at its Ser-347 and Ser-437 sites.

(A) A schematic diagram showing the full-length and truncated HbCAT2, including the N-terminal (residues 1 –247) and C-terminal (residues 248–492) fragments.

(**B**) Yeast two-hybrid assay showing the interaction of HbSnRK2.6A with the N-terminus (residues 1–247) and the C-terminus (residues 248–492) of HbCAT2.

(**C**) *In vitro* phosphorylation of wild-type and mutated HbCAT2 proteins by HbSnRK2.6A. The purified His-HbSnRK2.6A was incubated with GST-tagged HbCAT2, HbCAT2-S9A, HbCAT2-T105A, HbCAT2-S347A, HbCAT2-S437A, or HbCAT2-S347A/S437A proteins at room temperature for 30 min in the presence or absence of ATPγS. The protein mixture was alkylated and then separated by SDS-PAGE gel. The phosphorylation signals were detected by the anti-thiophosphate ester antibody.

(**D**) The catalase activities of the purified GST-tagged HbCAT2, HbCAT2-S9A, HbCAT2-T105A, HbCAT2-S347A, HbCAT2-S437A, or HbCAT2-S347A/S437A proteins. Data are means ± SD (n = 3). Bars with different letters indicate significant differences at $P < 0.05$, determined using ANOVA with a Tukey's multiple comparison test.
