# Supplementary material for: Transcription and phosphorylation of CATALASE2 contribute to cold stress tolerance in Hevea brasiliensis
Source: Plant J. 2026 Aug 28;127(4):e71097. doi: 10.1111/tpj.71097 (PMC13522878; doi:10.1111/tpj.71097)
Supplement: Supplementary file 4 [file TPJ-127-0-s004.pdf]

Supplemental Table S2. Gene sequences used in this study.

Gene name	Sequence (5' to 3')
HbCAT2 CDS	ATGGATCCCTACAAGAACCGTCCATCAAGCGCTTACGATTCCCCCTTCTGGACTACGAATTACGGTGCTCCAGTAT<br>GGAACAACAATTCGTCCTTGACTGTTGGGCCCAGAGGTCCGATCCTTCTTGAAGACTATCATCTGGTGGAGAAAC<br>TTGCGAACTTTGATAGGGAGAGGATTCCAGAGCGTGTTGTCCATGCTGGGGGAGCAAGTGCAAAGGGTTTCTT<br>TGAGGTCACACATGATGTCTCTGACCTTACATGTGCTGATTTCCTTCGAGCCCCAGGGGTTCAGACTCCTGTCATT<br>GTCCGTTTCTCCACTGTTATTCATGAGCGGGGCAGCCCAGAAACCATAAGGGATCCTCGTGGTTTTGCTGTGAAG<br>TTTTACACCAGAGAGGGCAACTTTGATCTGGTGGGAAACAATTTCCCTGTTTTTTTTATCCGTGATGGAATAAAGT<br>TCCCAGATTTGATACATGCATTTAAGCCCAACCCTAAGTCTCACATCCAGGAGAGCTGGAGGATCTTTGACTTCCT<br>ATCCCATGTTCCTGAAAGCTTGCATATGCTCAGCTTCCTTTTTGATGATTTGGGTATTCCACAAGATTACCGGCACA<br>TGGAAGGCTCTGGTGTTAACACCTATACTCTAATCAACAAGGCTGGAAAAGTACACTATGTGAAATTTCACTGGA<br>AACCTACTTGTGGTGTGAAGTGTTTCCTGGAGGAAGAGGCAGTCAAGGTTGGAAGAACCAATCATAGCCATGCC<br>ACCCAGGATCTGTATGACTCCATTGCAGCTGGCAACTATCCAGAGTGGAAGCTTTACATCCGAACAATGGACCCC<br>AATCATGAAGACAAATTTGACTTTGATCCACTTGATGTGACTAAGACCTGGCCTGAGGATATCCTGCCTCTGCAGC<br>CAGTTGGCCGCTTGGTCTTGAACAAGAACATTGACAATTTCTTTGCTGAGAATGAGCAACTTGCATTCTGCCCGG<br>GTATTGTGGTCCCAGGTGTTTCATATTCGGATGATAAATTACTTCAGACTAGGATCTTTTCCTATTCTGATACCCAGA<br>GGCACCGACTTGGACCAAACTATCTGCAGCTCCCTGTTAATGCTCCAAAGTGTGCTCATCATAACAATCACCATGA<br>CGGTTTCATGAATTTCATGCACAGGGATGAGGAGGTCAATTACTTCCCGTCGAGGTATGATCCAGTTCGCCATTCT<br>GAGAGGTTCCCCATTCCTTCAGCCATTTGCAGTGGAAAGCGCGAGAAGTGTGTCATTGAGAAGGAGAACAATTT<br>CAAGCAACCTGGAGAGAGATATCGGTCTTGGGCACCTGACAGGCAAGAGCGATTCCTACGTCGGATGGTCGAG<br>TGCTTATCTGACCCCCGTATCACTCACGAGCTTCGTAGTATCTGGATCCCCTACTGGACTGAGTGTGACAGATCTCT<br>GGGTCAAAAGCTAGCATCTCGTCTCAATGCGAGGCCAAGCATTTGA
HbCAT2 promoter	TGAGATTGAGTGTTCGAGCAGTATTAGTTCTAATCTATTGAGTTAGGGATATTGTACAATATCCAAAAAATGCATTT<br>AAATTATGTAATATAATTTTATTTTAAAAAAATTATAAAATTTTAAAAAAATATCTTATCTCTGTAACTAGGGATAACT<br>GATAATAGTCTTATTTAAATTTATAATTAAATTAAATCAATTTAAATTTAAAATTATATTAAAATCAATGATCTTAAATT<br>ACATAAAATTGGATAAATTAAAATTAAATTGAAAAAAAAAACTAAAATTAGAATTGAAACTCTTTTGGTGTAAAAT<br>TAGAATCGATCTGTGCAGGTGAGGTTTTTTTTATATAATCAAAAAATAAAATAATTATAATAAATAGTCTTTATTTTAT<br>TAAGTTTGAGATTTAAAATGACGTATCCAATTTAATCAAATCACATAAAAAGATATTTATTATTAAATTTGAAGTGAT<br>ATTCAATAAGTCGAATTATTAAATAATTTATTATTATTTATTATTATATTTTTAAATAAAAATAATATTTAATTATTATATT<br>AATATAAAAAATTAAAAATTAAAATTTAGATTAACCACTAAATTAGTATACACTCGGTGCTTTTGTAGGTGAGCTTA<br>CCTCACACTCACAGCCCATTCTGATCCTTATCATCAAAATGATCAATTTTAGCAGCTTTCGCACTCTCTATCGCTATC<br>TCTTTAATATCCGAAACTATCCATCTTCAGCACTTTTACATCGTATTAGGGCACACGTTTCAAGGCTTATTATGATAA<br>CGTGTCAAAATATCAGTGGCCCACGTGGCCTTATGTGGGTTTAAACTTAGATAAGGAGCCATTACCTGGTGACGT<br>TTTCAATTGTTTTATTACTATTTTAGTATTTGATTTTCCCAAATCCCAATTCCTCTCCGATCAGTCTTGATTTTCCCAC<br>CACATTTAAATCCTGATGCCGTAGTGGGTCCCACTTCTTCTCTTTCCTTTTCATCCAGTCCTGCATAAATATCGTCGT<br>GCTTTCAATTTATCGCGAATTGTGGCAGAGCCAATGTTCATCTTTAACCATTCAATTTATCCACCTGTCTTCATTTTA<br>GGGAGAAATTCATCTAAAATTTAATAAAATAATAATAAATAAAGTTTTTTTTAAATTTTTGACATTAAATATTGATATT<br>TTTCTATATCGAGTCCATTATATTTTATGTATTTAACAAAAAATTAAAAAAAATCTCATTATTCTCGATAGTATATATTC<br>AAATAGAAGCATTATTGTAATCTTCAATTTAATGATTCAAAATTTTTGTGAGTTTTTCTTAATTTAAGTAAGAAATAT<br>TTTTAATTAAATAGTGGATTATTTTTCTAAATAAAATAGAATTTTTATAATAATATTATTTACAAATTATAAAATTTTTAT<br>TTAAATTAAAATTAAAACTATTAATGTTATAAATAGAAATACTTCAATATAATTAATATTTTTATATATTTATTTATTTATA<br>ATAAGTTTATTATTTTATGCAAACTATGACAACGTGCTGTACAATTTCACTTGTGGCTGTTTTTTATCGTCTAAACGT<br>ATATATCGCGACAAGCGTAAAACAAGGCGAGATGTGAAATTACCGACAGTAAAATTTTAAATTTATCTGCATGGGA<br>GAAAAAATTTTACATTTTTGGGAATATTTTTTTTATTTTTTATTTTTATGGATAACCATAGGCATTACAAGAAAATGG<br>ACGTGCAAGCGACGAGCCTCTATAAATAGCCAGAAAGTAGAGTTCTTCTTCGAATTGAACGCGACTCTCACATTT<br>AATTCGGTGTTTTGGGGTCTCCTTTTCTCACCAAACCCATCAAAAATTCACAAATATCTCTTGCT
HbSnRK2.6A CDS	ATGGATCGTTCGGCGGTTACCGTAGGTCCCGGTATGGATATGCCGATTATGCACGATAGTGACCGTTACGAGCTG<br>GTGCGAGATATCGGGTCTGGGAATTTCGGAGTGGCGAGATTGATGAGAGATAAGCAGACTGACGAGCTCGTTG<br>CCGTCAAGTATATCGAGAGAGGTGAGAAGATAGATGAAAATGTACAAAGGGAAATTATTAACCACAGGTCATTGA<br>GGCATCCTAACATTGTCAGATTCAAAGAGGTCATATTAACACCAACACACCTGGCTATTGTGATGGAGTATGCATC<br>TGGTGGAGAACTTTTTGAGCGGATATGCAATGCAGGCCGCTTCAGTGAAGATGAGGCACGCTTTTTCTTCCAAC<br>AACTTATATCGGGAGTTTGCTACTGCCATGCAATGCAAGTATGTCACCGTGACTTAAAATTGGAGAATACGTTGTT<br>GGATGGAAGCCCCGCACCTCGTCTTAAGATTTGTGACTTTGGTTACTCTAAGTCATCTGTGCTCCATTCACAACCA<br>AAATCTACTGTTGGAACTCCTGCATATATTGCACCTGAAGTGTTACTTAAGAAGGAATATGATGGCAAGATTGCAG<br>ATGTATGGTCTTGTGGGGTAACTTTATACGTAATGTTGGTGGGAGCATATCCTTTTGAGGACCCTGAGGAGCCTAA<br>GAATTTTCGCAAGACAATACATCGAATTTTGAATGTCCAGTACTCCATTCCTGACTATGTTCATATATCTCCTGAGTG<br>CCAGCACTTGATCTCAAGAATATTTGTGGCTGATCCTGCAAAGAGGATAAGCATACCTGAGATTAGGAACCACGA<br>ATGGTTTCTGAAGAATCTTCCAGCAGATCTCATGGATGTAAATACAATGAACAACCAGTTTGAAGAGCCTGATCTG<br>CCCATGCAGAGCATTGATGAAATAATGCAGATAATTTCTGAAGCCACCATCCCAGCCGCTGGTGCCCGTGGTCTC<br>AATCAATATCTGACTGGCAGCCTGGACATTGATGATGACATGGAGGAGGATCTAGAGAGTGATCCTGAACTTGAC<br>ATGGATAGTAGTGGAGAGATAGTATATGCAATGTGA
HbCBF1 CDS	ATGGATGTTTTCCCTCAATATTCTGACTCACTTCCTTTTGCCACTCATTCCTGCTCACTTCACTACCCAGAATCTTCT<br>ACATTGTCTGATACCTGCAGTGCTCTTCGTGCGAACCTTTCCGATGAGGAAGTCTTGTTAGCTTCCAGCTATCCCA<br>AGAAGCGCGCAGGCAGAAAGAAGTTCCGGGAGACACGCCATCCTATTTATCGTGGGGTCCGTCGGAGGAACTC<br>CGGGAAGTGGGTTTGTGAAATTAGAGAACCCAACAAGAAATCAAGAATATGGTTGGGGACTTTCCCCACGGAG<br>GAAATGGCTGCTAGGGCCCACGACGTGGCGGCGCTGGCTCTCAGAGGACGGTCTGCCTGCTTGAATTTTGCGG<br>ACTCGTCATGGCGACTTCCTGTACCTGCGTCCAGAGAAGCAAAGGACATTAGGAAGGCAGCAGCAGAGGCGGC

	GATGGCGTTCCAGCCGGAGGGGACAGAAGGGTTTTCTGGAGAATTAAAACAGGAAAATAAGTGGACGACAGA<br>GTCAGCGCCGGAGGATGTGTTTTATATGGACGAAGAGGCGGTTTTTGCAATGCCGGGACTACTGGCATCTATGG<br>CAGAAGGGATGCTACTTCCTCCACCTCAGTGCGTTGCAGGGAGCGGAGGAGAAGACGGAGAAATGGATGCAG<br>CTGATGTGTCACTATGGAGTTTTTCTATATAA
AtCAT2<br>CDS	ATGGATCCTTACAAGTATCGTCCAGCTAGTTCTTACAACTCTCCCTTCTTCACCACCAACTCTGGTGCTCCTGTATG<br>GAACAACAACTCCTCCATGACCGTTGGACCCAGAGGTCCTATCCTTCTTGAGGATTACCATCTCGTTGAGAAGCT<br>TGCCAATTTCGACAGGGAACGGATTCCAGAGCGTGTGGTTCATGCCAGAGGAGCCAGTGCTAAAGGTTTCTTTG<br>AGGTCACTCATGATATCTCTAACCTCACTTGTGCTGACTTTCTCCGAGCTCCCGGTGTTCAGACTCCTGTCATTGTC<br>CGGTTCTCCACCGTTATCCATGAGCGTGGAAGTCCCGAGACCTTGAGAGACCCTCGTGGTTTTGCAGTCAAGTTC<br>TACACCAGAGAGGGGAACTTTGATCTTGTTGGAAACAACTTTCCTGTTTTCTTCATCCGCGATGGGATGAAGTTC<br>CCTGACATGGTCCACGCTCTTAAGCCGAACCCAAAATCTCACATCCAAGAGAACTGGAGAATCCTTGACTTCTTC<br>TCCCACCACCCTGAAAGTTTGAACATGTTCACTTTCCTCTTCGATGATATCGGTATCCCACAAGATTACAGGCACAT<br>GGATGGTTCAGGTGTCAATACATACATGTTGATCAACAAAGCTGGCAAAGCTCACTACGTGAAGTTCCATTGGAA<br>ACCAACTTGTGGAGTCAAGTCTCTTTTGGAAGAAGATGCAATTCGTGTTGGAGGAACCAACCACAGTCATGCGA<br>CTCAAGACTTGTATGACTCTATAGCTGCTGGAAACTACCCTGAATGGAAGCTCTTTATCCAAATCATTGATCCTGCT<br>GATGAAGACAAGTTCGACTTTGACCCGCTCGATGTGACCAAGACCTGGCCTGAGGATATCTTGCCTCTTCAACCT<br>GTTGGACGTATGGTGTTGAACAAGAACATTGACAACTTCTTTGCAGAGAATGAGCAACTTGCTTTCTGTCCTGCA<br>ATTATTGTCCCAGGGATACATTACTCAGATGATAAGCTGCTTCAAACCCGTGTCTTCTCCTATGCCGATACTCAGAG<br>ACACCGTCTTGGACCAAACTACCTTCAGCTGCCAGTCAATGCTCCAAAATGTGCTCACCACAACAACCACCATGA<br>GGGATTCATGAATTTCATGCACAGGGACGAGGAGGTTAACTACTTCCCGTCGAGGTATGACCAGGTTCGTCATGC<br>TGAGAAGTATCCAACTCCGCCTGCTGTCTGTTCTGGAAAACGTGAGAGGTGCATTATTGAGAAAGAGAACAACT<br>TCAAGGAGCCTGGAGAGAGATACCGTACCTTTACACCAGAGAGGCAAGAACGATTCATCCAGAGATGGATTGAT<br>GCCCTATCCGACCCACGCATCACGCATGAAATCCGCAGTATCTGGATCTCTTACTGGTCTCAGGCTGATAAGTCTTT<br>GGGACAGAAGCTGGCAAGCCGTCTGAACGTGAGACCAAGCATCTAA
AtSnRK2.6A<br>CDS	ATGGATCGACCAGCAGTGAGTGGTCCAATGGATTTGCCGATTATGCACGATAGTGATAGGTATGAACTCGTCAAG<br>GATATTGGCTCCGGTAATTTTGGAGTTGCGAGATTGATGAGAGACAAGCAAAGTAATGAGCTTGTTGCTGTTAAA<br>TATATCGAGAGAGGTGAGAAGATAGATGAAAATGTAAAAAGGGAGATAATCAACCACAGGTCCTTAAGACATCC<br>CAATATCGTTAGATTCAAAGAGGTTATATTAACACCAACCCATTTAGCCATTGTTATGGAATATGCATCTGGAGGAG<br>AACTTTTCGAGCGAATCTGCAATGCAGGCCGCTTCAGCGAAGACGAGGCGAGGTTTTTCTTCCAGCAACTCATT<br>TCAGGAGTTAGTTACTGTCATGCTATGCAAGTATGTCACCGAGACTTAAAGCTCGAGAATACGTTATTAGATGGTA<br>GCCCGGCCCCTCGTCTAAAGATATGTGATTTCGGATATTCTAAGTCATCAGTGTTACATTCGCAACCAAAATCAACT<br>GTTGGAACTCCTGCTTACATCGCTCCTGAGGTTTTACTAAAGAAAGAATATGATGGAAAGGTTGCAGATGTTTGG<br>TCTTGTGGGGTTACTCTGTATGTCATGCTGGTTGGAGCATATCCTTTCGAAGATCCCGAGGAACCAAAGAATTTCA<br>GGAAAACTATACATAGAATCCTGAATGTTCAGTATGCTATTCCGGATTATGTTCACATATCTCCTGAATGTCGCCATT<br>TGATCTCCAGAATATTTGTTGCTGACCCTGCAAAGAGGATATCAATTCCTGAAATAAGGAACCATGAATGGTTTCT<br>AAAGAATCTACCGGCAGATCTAATGAACGATAACACGATGACCACTCAGTTTGATGAATCGGATCAACCGGGCCA<br>AAGCATAGAAGAAATTATGCAGATCATTGCAGAAGCAACTGTTCCTCCTGCAGGCACTCAGAATCTGAACCATTA<br>CCTCACAGGAAGCTTGGACATAGATGACGATATGGAGGAAGACTTAGAGAGCGACCTTGATGATCTTGACATCG<br>ACAGTAGCGGAGAGATTGTGTACGCAATGTGA
